# In vitro interactions of *Alternaria* mycotoxins, an emerging class of food contaminants, with the gut microbiota: a bidirectional relationship

**DOI:** 10.1007/s00204-021-03043-x

**Published:** 2021-04-13

**Authors:** Francesco Crudo, Georg Aichinger, Jovana Mihajlovic, Elisabeth Varga, Luca Dellafiora, Benedikt Warth, Chiara Dall’Asta, David Berry, Doris Marko

**Affiliations:** 1 Department of Food Chemistry and Toxicology, University of Vienna, Währinger Str. 38, 1090 Wien, Austria; 2 Department of Food and Drug, University of Parma, Parco Area delle Scienze 27/A, 43124 Parma, Italy; 3 Department of Microbiology and Ecosystem Science, Centre for Microbiology and Environmental Systems Science, University of Vienna, Althanstr. 14, 1090 Vienna, Austria; 4 Joint Microbiome Facility of the Medical University of Vienna and the University of Vienna, Vienna, Austria

**Keywords:** Microbiome, Chemical mixture, Lipophilicity, Adsorption, Biofilm

## Abstract

The human gut microbiota plays an important role in the maintenance of human health. Factors able to modify its composition might predispose the host to the development of pathologies. Among the various xenobiotics introduced through the diet, *Alternaria* mycotoxins are speculated to represent a threat for human health. However, limited data are currently available about the bidirectional relation between gut microbiota and *Alternaria* mycotoxins. In the present work, we investigated the in vitro effects of different concentrations of a complex extract of *Alternaria* mycotoxins (CE; containing eleven mycotoxins; e.g. 0.153 µM alternariol and 2.3 µM altersetin, at the maximum CE concentration tested) on human gut bacterial strains, as well as the ability of the latter to metabolize or adsorb these compounds. Results from the minimum inhibitory concentration assay showed the scarce ability of CE to inhibit the growth of the tested strains. However, the growth kinetics of most of the strains were negatively affected by exposure to the various CE concentrations, mainly at the highest dose (50 µg/mL). The CE was also found to antagonize the formation of biofilms, already at concentrations of 0.5 µg/mL. LC–MS/MS data analysis of the mycotoxin concentrations found in bacterial pellets and supernatants after 24 h incubation showed the ability of bacterial strains to adsorb some *Alternaria* mycotoxins, especially the key toxins alternariol, alternariol monomethyl ether, and altersetin. The tendency of these mycotoxins to accumulate within bacterial pellets, especially in those of Gram-negative strains, was found to be directly related to their lipophilicity.

**Supplementary Information:**

The online version contains supplementary material available at 10.1007/s00204-021-03043-x.

## Introduction

Through its metabolic activities and interactions with the host, the complex microbial community that inhabits the human intestine, termed the “gut microbiota”, plays a key role in the well-being of humans (Clemente et al. 2012; Jandhyala et al. 2015). Any factor able to modify the composition and activity of the gut microbiota might influence both normal physiology and disease susceptibility of its host (Hasan and Yang 2019). Among the various factors influencing the gut microbial composition, diet is the most important one (Scott et al. 2013). In addition to the nutritional composition of ingested foods, the presence of food contaminants, such as mycotoxins, can affect the balance within the intestinal microbiome (Liew and Mohd-Redzwan 2018).

Mycotoxins are fungal toxic metabolites that may enter the food chain due to the ability of toxigenic fungi to infest a wide variety of crops and food commodities (Berthiller et al. 2013; Drejer Storm et al. 2014). Out of the roughly 400 mycotoxins known to date, only a few are regulated (Righetti et al. 2016). Among the compounds currently under consideration for new regulatory limits in the European Union, toxins produced by the genus *Alternaria* are gaining increasing interest due to the high level of occurrence in fresh and processed foods (e.g., fruits, vegetables, cereals, nuts, seeds and oils) and because of the toxic effects observed in vivo (e.g., teratogenic and fetotoxic effects) and in vitro (e.g., androgenic, estrogenic, genotoxic, mutagenic, and clastogenic effects) (Crudo et al. 2019). *Alternaria* species can produce more than 70 toxins, among which, alternariol (AOH), alternariol monomethyl ether (AME), altenuene (ALT), tenuazonic acid (TeA), tentoxin (TEN), alterperylenol/alteichin (ALP), and the altertoxins (ATXs) I and II, are the best characterized (Crudo et al. 2019). Of note, the dibenzo-α-pyrones AOH and AME, and the epoxide-carrying compounds ATX-II and stemphyltoxin III (STTX-III) were reported to induce DNA strand breaks in vitro and to act as topoisomerase poisons and topoisomerase inhibitors, respectively (Fehr et al. 2009; Fleck et al. 2016). AOH and AME also showed clastogenic, mutagenic and estrogenic effects (Brugger et al. 2006; Lehmann et al. 2006; Dellafiora et al. 2018), as well as the ability to modulate innate immunity (Grover and Lawrence 2017; Kollarova et al. 2018). Information about the absorption, distribution and excretion of *Alternaria* toxins is currently available only in mice (Schuchardt et al. 2017) and rats (Puntscher et al. 2019a), while it is still scarce in humans. Consequently, the real impact on human health has yet to be clarified. A recent in vivo study in rats showed a fecal excretion > 89% for AOH and AME, while the perylene quinones ATX-I and ALP were recovered only at very low levels (< 5%) (Puntscher et al. 2019a). Interestingly, the highly reactive epoxide-carrying compounds ATX-II and stemphyltoxin III (STTX-III) were not recovered at all (Puntscher et al. 2019a). On this basis, the high level of fecal excretion found for AOH and AME suggests that potential toxic effects are likely to occur predominantly at the level of the intestinal tract rather than systemically. Thus, possible inhibitory or growth-promoting effects on members of the gut microbiota cannot be excluded. On the other hand, the low recovery of ATX-I, ATX-II, STTX-III, and ALP might be the consequence of chemical transformation occurring at the gastro-intestinal level. Considering the ability of the human gut microbiota to participate in the detoxification processes of xenobiotic compounds (Collins and Patterson 2020), the involvement of intestinal bacteria in the mechanisms of removal of ingested mycotoxins must be hypothesized, especially in view of a possible risk assessment of *Alternaria* mycotoxins.

A bi-directional interaction between mycotoxins and the gut microbiota has been reported by several authors, who investigated the effects exerted by mycotoxins on the gut microbiota and the ability of gut microbes to bind or metabolize mycotoxins ingested with the diet. As an example, germ-free rats inoculated with fecal microbiota from healthy humans showed a significant decrease of the concentration levels of *Escherichia coli* and an increase of the Bacteroides/Prevotella group after treatment with the *Fusarium* mycotoxin deoxynivalenol (DON) (Saint-Cyr et al. 2013). On the other hand, the ability of microorganisms from the intestines of broilers to transform DON to the less toxic de-epoxy-DON was also reported (He et al. 1992; Yu et al. 2010). Another mycotoxin that was shown to perturb the gut bacterial population is T2-toxin, which led to an increase of the aerobic bacteria in the intestines of rats and swine (Tenk et al. 1982). Microorganisms from human feces were found to release the parental compound from the glucoside metabolites of T2-toxin, thus contributing to the increase of the amount of free absorbable mycotoxin (Gratz et al. 2017). The transformation of the mycotoxin zearalenone (ZEN) into unknown metabolites (Gratz et al. 2017), as well as its ability to modify the microbial diversity in porcine ascending colon contents, has also been reported (Piotrowska et al. 2014). A modulation of microbiota composition was also found to be induced by ochratoxin A and aflatoxin B1 (Baines et al. 2013; Ouethrani et al. 2013; Guo et al. 2014; Wang et al. 2016), as well as by mixtures of fumonisins B1 + B2 (Burel et al. 2013) and ZEN + DON (Piotrowska et al. 2014).

We recently summarized the available data on the co-occurrence of *Alternaria* mycotoxins in food, highlighting the high frequency of co-occurrence of multiple metabolites (Crudo et al. 2019). Based on this, a complex extract of *Alternaria alternata* cultured on rice, containing eleven chemically characterized mycotoxins, was used in the present work to investigate the bidirectional relation existing between *Alternaria* mycotoxins and the gut microbiota. The aim of this work was to evaluate the effects exerted by the complex mixture of *Alternaria* mycotoxins on human gut bacterial strains belonging to five different phyla. To investigate the possible contribution of bacteria to the reduction of their harmful effects, the ability of the same bacterial strains to metabolize or adsorb *Alternaria* mycotoxins was studied.

## Materials and methods

### Complex extract of *Alternaria* mycotoxins (CE)

The mycotoxin extract used in the present study is a complex mixture of *Alternaria* mycotoxins obtained in our laboratory by growing the *Alternaria alternata* strain DSM 62,010 on long rice with a subsequent extraction procedure, as previously described (Puntscher et al. 2019b). The obtained extract was chemically characterized by LC–MS/MS. Mycotoxin concentrations in the extract are listed in Table 1 and adapted from Aichinger et al. (2019). The chemical structures of mycotoxins contained in the extract are shown in Online Resource 1.

**Table 1:** Composition of the *Alternaria* mycotoxin extract characterized by LC–MS/MS analysis (adapted from Aichinger et al. 2019)

*Alternaria* mycotoxins (Abbreviations)	Concentration (mg toxin/g extract)
Alternariol (AOH)	0.79
Alternariol monomethyl ether (AME)	0.65
Altenuene (ALT)	0.78
Tenuazonic acid (TEA)	597
Tentoxin (TEN)	0.02
Altertoxin I (ATX-I)	9.92
Altertoxin II (ATX-II)	14.1
Alterperylenol (ALP)	12.6
Stemphyltoxin III (STTX-III)	21.0
Altenusin (ALS)	0.28
Altersetin (AST)	18.4

### Chemicals for sample preparation and LC–MS/MS analysis

Methanol and acetonitrile were acquired from Honeywell (Seelze, Germany), water was purchased from VWR International GmbH (Vienna, Austria), while ammonium acetate and 25% ammonia solution in water were obtained from Sigma–Aldrich Handels GmbH (Vienna, Austria). All solvents were of LC–MS grade. Reference materials of *Alternaria* toxins were kindly provided by other researchers or purchased from several suppliers. For further details, refer to Puntscher et al. (2019b).

### Bacterial strains and culture media

The human gut bacterial strains were acquired from culture collections or isolated from human feces in our laboratory and identified through the 16S rRNA gene sequencing. Detailed information about the strains and media used in this study can be found in Online Resource 2. Fourteen bacterial strains were chosen to have at least one representative strain for each of the most dominant gut microbial phyla (i.e. Firmicutes, Bacteroidetes, Actinobacteria, Proteobacteria, and Verrucomicrobia) and to test both Gram-negative and Gram-positive strains. *Bacteroides caccae* (BC), *Bacteroides eggerthii* (BE), *Bacteroides thetaiotaomicron* (BT), *Bacteroides vulgatus* (BV), *Parabacteroides distasonis* (PD), *Escherichia coli* (EC), and *Clostridium innocuum* (CI), were cultivated in a supplemented brain heart infusion broth (BHI-S, ATCC Medium 1293), while for *Alistipes finegoldii* (AF), *Alistipes timonensis* (AT), and *Ruminococcus bicirculans (RB)* a modified YCFA broth (Yeast extract-Casein hydrolysate-fatty acids) supplemented with glucose was employed (YCFA-g, DSMZ Medium 1611). For the growth of *Akkermansia muciniphila*, brain hearth infusion broth (Oxoid; CM1135) supplemented with 0.05% pig gastric mucin type II (Sigma-Aldrich, M2378) was prepared (BHI-muc). A modified MRS broth (De Man, Rogosa, Sharpe; m-MRS) was used for the growth of *Lactobacillus hominis, Bifidobacterium longum* and *Bifidobacterium *sp. The m-MRS broth was composed as follows (per liter): 100 g Tryptone (Oxoid; LP0042), 10 g beef extract (Sigma-Aldrich; B4888), 5 g yeast extract (Oxoid; LP0021), 20 g glucose (Oxoid; LP0071), 1 g tween 80 (Fluka; 93,780), 2 g K_2_HPO_4_ (Merck; 1.05104.1000), 5 g Na-acetate (Sigma-Aldrich; S8750), 0.2 g MgSO_4_ (Sigma-Aldrich; M2643). Prior to performing an experiment, media were left at least for 20 h in the anaerobic tent (5% CO_2_, 5% H_2_ and 90% N_2_) to allow deoxygenation.

### Determination of the minimum inhibitory concentration (MIC)

Determination of the MIC values of the *Alternaria* mycotoxin extract was preliminarily performed on *B. thetaiotaomicron*,* B. vulgatus*,* B. caccae*,* C. innocuum* and *E. coli* with the aim to investigate the ability of the extract to completely inhibit the growth of the strains and define the concentrations to be tested for the subsequent analysis. Briefly, strains were grown overnight in BHI-S medium under anaerobic conditions. Then, the optical density at 600 nm (OD_600_) was set to 0.1 and 50 µl of the adjusted cultures were pipetted in 96-well plates and diluted with 50 µl of the test media containing the various concentrations (2 × concentrated) of the CE dissolved in DMSO. Thus, the final OD_600_ was 0.05. For each CE concentration tested, wells containing the corresponding percentages of DMSO were also prepared as controls. The 96-well plates also included growth controls (media plus bacteria, without DMSO) and a negative control (media). The final CE concentrations tested were 200, 100, 50, 25, 12.5, 6.25, 3.13, 1.56 µg/mL (maximum DMSO concentration of 0.4%) and each concentration was tested in three biological replicates. Plates were incubated under anaerobic and static conditions for 24 h at 37 °C, followed by optical density measurements of the wells at 600 nm with a microplate reader (Multiskan™ GO Microplate Spectrophotometer, Thermo Scientific). The lowest concentration of CE which reduced bacterial growth by 90% or higher was considered to be the MIC value.

### Analysis of the growth curves

Determination of bacterial growth curves of the strains was carried out by measuring the optical density at 600 nm. Each strain was grown overnight in its appropriate medium at 37 °C under anaerobic conditions. Afterward, the overnight culture was diluted with the appropriate medium to a starting OD_600_ of 0.1, dispensed into wells of a sterile 96-well flat-bottomed microtiter plate (Costar 3595, Corning Inc., Corning, NY, USA) and diluted 1:1 with the test media containing the different concentrations of CE or DMSO (final volume of 200 µl/well). The final CE and DMSO concentrations tested were 50 µg/mL, 25 µg/mL, 5 µg/mL, 0.5 µg/mL, and 0.1%, 0.05%, 0.01%, 0.001%, respectively. The final OD_600_ was 0.05. The prepared plates, which also included negative controls (media) and growth controls (media plus bacteria, without DMSO), were wrapped with parafilm to prevent dehydration and incubated for 24 h at 37 °C in a microplate reader (Multiskan™ GO Microplate Spectrophotometer, Thermo Scientific) placed in an anaerobic tent. Plates were shaken continuously at low speed (5 Hz, amplitude 15 mm) during the incubation time and for 10 s at medium speed (11 Hz, amplitude 3 mm) prior to each read. The OD_600_ was recorded automatically every 20 min for a total of 24 h. Each condition was tested in at least three biological replicates. Quantification of the area under the experimental growth curves was carried out using the R package Growthcurver, available for installation from the Comprehensive R Archive Network (CRAN).

### Quantification of biofilm biomasses

Evaluation of the ability of the extract to affect the formation of biofilms produced by *E. coli*,* B. vulgatus*,* B. thetaiotaomicron*, and *B. caccae* was carried out according to the microtiter-plate test described by Stepanović et al. (2007) with slight modifications. Briefly, overnight cultures of the selected strains were diluted with their strain-specific media to an OD_600_ of 0.1. Then, 75 µl of the adjusted cultures was transferred into 96-well cell culture plates and diluted 1:1 with the test media containing the different percentages of DMSO (final concentrations of 0.1%, 0.05%, 0.01%, 0.001%) or concentrations of CE (final concentrations of 50 µg/mL, 25 µg/mL, 5 µg/mL, 0.5 µg/mL). The final OD_600_ was 0.05. Plates were incubated for 24 h or 48 h under anaerobic conditions and without shaking. Afterward, OD_600_ values were recorded to exclude bacterial growth inhibitions (Haney et al. 2018), plates were emptied, washed twice with tap water to remove planktonic cells and heat-fixed at 60 °C for 1 h. Biofilms were stained by adding 200 µl of 1% crystal violet solution to each well and incubating the plates at room temperature (RT) for 15 min. Then, plates were emptied, washed tree times with tap water and dried overnight at RT. Crystal violet was resolubilized by adding 200 µl 30% acetic acid. After 15 min incubation at RT, absorbance at 570 nm was measured by means of a microtiter plate reader (Multiskan™ GO Microplate Spectrophotometer, Thermo Scientific). Conditions for each strain were tested in at least three biological replicates. Definition of cut-off values, that allowed to distinguish biofilm-producing from non-biofilm-producing strains, was performed according to Stepanović et al. (2007) as follows: OD_cut-off_ = mean ODs of uninoculated medium + 3 times standard deviation.

### Sample preparation for LC–MS/MS analysis

After 24 h incubation of the strains with the various concentrations of CE, the bacterial suspension of each well was collected and immediately stored at − 80 °C. To investigate whether the bacterial strains were able to metabolize or adsorb mycotoxins of the *Alternaria* extract, samples treated with 25 µg/mL of CE were chosen to be analyzed by LC–MS/MS analysis. The choice to analyze samples from this concentration was made by evaluating both the detection limits of mycotoxins and the results obtained through the analysis of the bacterial growth curves. The aim was to analyze the samples whose CE concentration had caused only a minor or no effect on bacterial growth, in such a way as not to affect the metabolic activity of the bacteria tested. For the extraction of mycotoxins from supernatants, treated and control samples were thawed and centrifuged at 20,000 rcf for 10 min (4 °C) to pellet the bacteria. Then, the supernatants were transferred into new tubes, diluted 1:5 with an ice-cold extraction solvent (ACN/MeOH, 1:1, v/v), vortexed and placed at − 20 °C for 1 h to allow precipitation of proteins. Then, samples were centrifuged (20,000 rcf, 10 min, 4 °C) and the resulting supernatants were transferred into HPLC vials. As for the extraction from the bacterial pellets, after pelleting the bacteria through centrifugation (20,000 rcf, 10 min, 4 °C), a step of washing with phosphate buffer saline solution (PBS, 0.1 mol/L, pH 7.4) was performed to ensure the removal of mycotoxins present in the tube and deriving from the removed supernatants. After the PBS was removed, the washed bacterial pellets were resuspended with an extraction solvent (ACN/MeOH/water, 2:2:1, v/v/v), vortexed and sonicated for 15 min (on ice). After 1 h incubation at − 20 °C, precipitated proteins and bacteria were pelleted by centrifugation (20,000 rcf, 10 min, 4 °C) and supernatants transferred into HPLC vials. Extracted samples were immediately analyzed or stored at − 80 °C until analysis.

### LC–MS/MS analysis

The extracted pellets, supernatants, and controls were analyzed using a high-performance liquid chromatographic system (UltiMate3000, Dionex Thermo Fisher Scientific) coupled to a TSQ Vantage triple quadrupole mass spectrometer equipped with a heated electrospray ionization interface (Thermo Fisher Scientific).

The LC–MS/MS method used in the present study has been already used in our recent work for the quantification of *Alternaria* mycotoxins in human fecal slurries (Crudo et al. 2020). Briefly, a Supelco Ascentis^®^ Express C18 column (100 × 2.1 mm, 2.7 µm) equipped with a pre-column (SecurityGuard™, C18, 2 mm, Phenomenex, Torrance, CA, USA) was employed for the chromatographic separation. An ammonium acetate in water solution (5 mM, pH adjusted to 8.7 with a 25% NH_4_OH solution) and MeOH were used as eluents. MS data for the extracted samples were acquired in multiple reaction monitoring mode, applying negative electrospray ionization. Further information about the applied LC–MS/MS method can be found in Puntscher et al. (2018). External calibration was employed as a quantification technique and injection of the calibration set was performed after every 20–30 samples. Monitoring of the instrumental conditions and the data acquisition were carried out using the software package Thermo Xcalibur™ (v. 4.0.27.42, Thermo Scientific), while TraceFinder™ software (v. 3.3; Thermo Scientific) was employed for data evaluation.

### In silico prediction of mycotoxins lipophilicity

The prediction of mycotoxins lipophilicity was performed using the freely available online tool SwissADME (http://www.swissadme.ch). The “consensus log *P*_o/w_ value” for each mycotoxin, which is the arithmetic mean of the values obtained by five computational methods, was used for the LC–MS/MS data evaluation.

### Statistical analysis

Significant differences (*p* < 0.05 or *p* < 0.01) between control and treated samples were evaluated by Independent Student *t *test, performed using SPSS software (v. 23.0, SPSS Inc., Chicago, IL, USA). Principal component analysis of the LC–MS/MS data was carried out using OriginPro software (v. 2018, OriginLab Corporation, Northampton, MA, USA).

## Results

### Effects of *Alternaria* mycotoxins on bacterial strains

#### Minimum inhibitory concentration (MIC)

The complex *Alternaria* extract (CE), containing eleven characterized mycotoxins (Table 1), was tested against the human gut species *Bacteroides thetaiotaomicron*,* Bacteroides vulgatus*,* Bacteroides caccae*,* Escherichia coli*, and *Clostridium innocuum* to evaluate its ability to inhibit the growth of the strains. Results from the test showed the inability of the extract to inhibit the growth of *B. thetaiotaomicron*,* B. caccae*, and *E. coli* at any tested concentration of the *Alternaria* extract (ranging from 1.56 to 200 µg/mL). In contrast, MIC values of 50 µg/mL and 100 µg/mL were found for *B. vulgatus* and *C. innocuum*, respectively.

#### Influence on bacterial growth kinetics

**Fig. 1:**
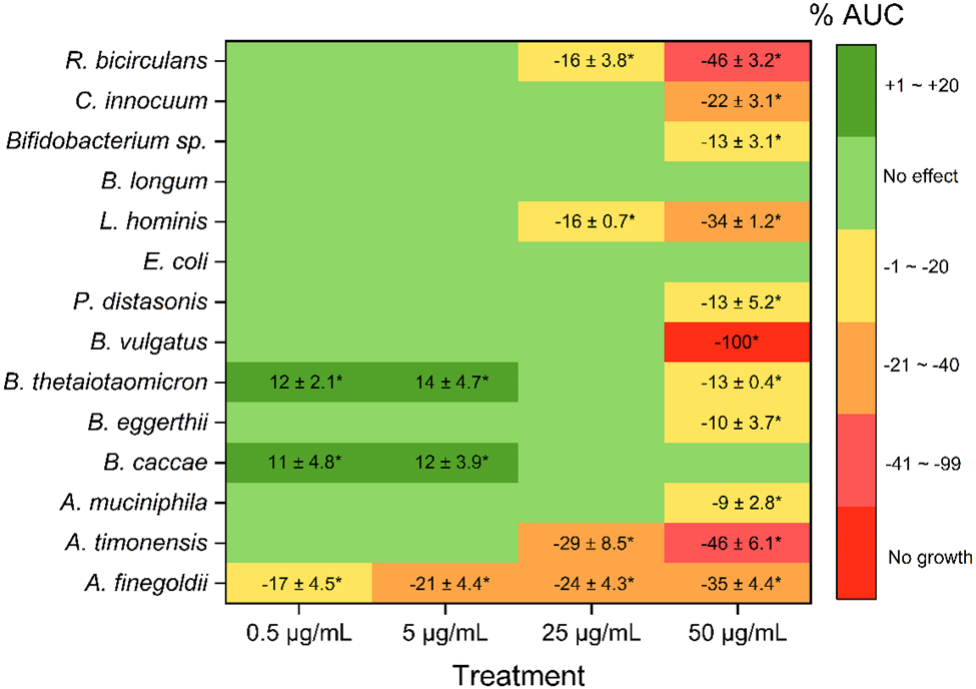
Modifications of the area under the curve (AUC) of growth curves induced by 24 h incubation of bacterial strains with different concentrations of the *Alternaria* extract. Reported numbers refer to mean ± SD of increase or reduction of AUC compared to the respective control (strain + DMSO). Percentages of DMSO varied from 0.1% to 0.001% (for treatments with 50 µg/mL or 0.5 µg/mL of CE, respectively). Each condition was tested in triplicate. Significant differences to the DMSO control were evaluated by Student’s *t* test (*p < 0.05)

**Fig. 2:**
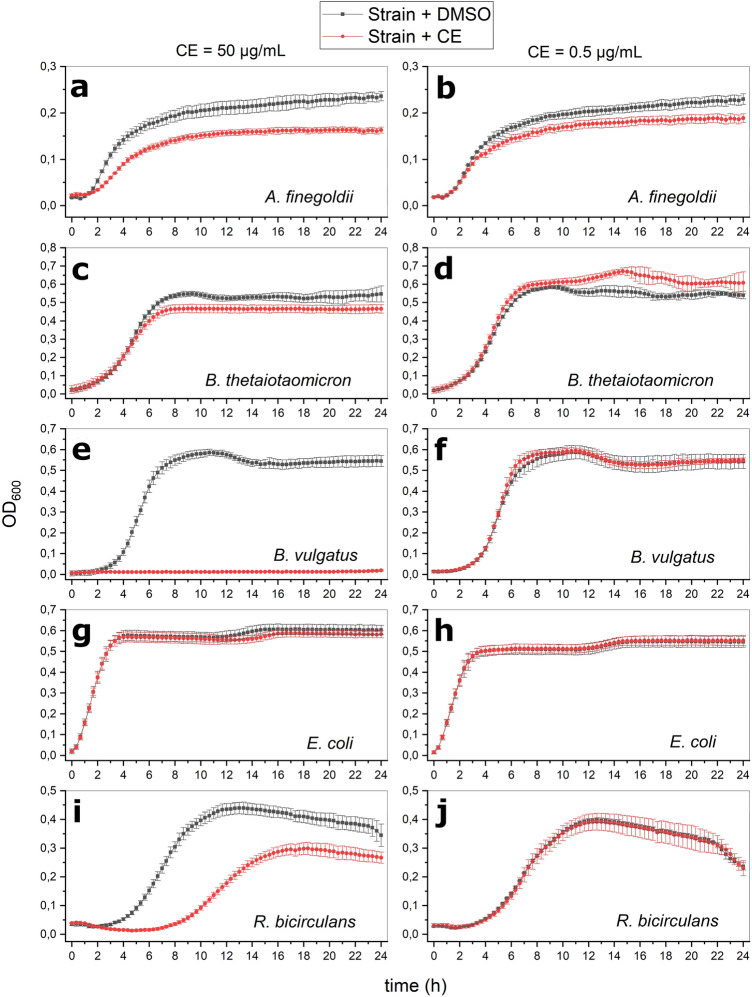
Representative growth curves expressed as optical density measured at 600 nm of different bacterial strains during treatment with the maximum (50 µg/mL, left column) and minimum (0.5 µg/mL, right column) concentration of the *Alternaria* extract (CE). Bacterial growth curves colored in black or red refer to the DMSO-treated or CE-treated strains, respectively

The ability of the *Alternaria* extract to affect the growth kinetics of 14 reference human gut bacterial strains was evaluated by monitoring the optical density of microbial suspensions treated with 0.5, 5, 25, and 50 µg/mL of CE for 24 h. Bacterial growth curves of all fourteen strains treated with the various concentrations of CE are shown in Online Resource 3. As summarized in Fig. 1, while increased or decreased of area under the curve (AUC) were found for most of the bacterial strains after exposure to specific CE concentrations (regardless of the Gram-type), the growth of some strains was not affected even after treatment with the highest CE concentration. Examples of the most diverse behaviors observed after exposure to the maximum and minimum concentrations of CE are reported in Fig. 2, where the growth curves of *Alistipes finegoldii* (2A, 2B), *B. thetaiotaomicron* (2C, 2D), *B. vulgatus* (2E, 2F), *E. coli* (2G, 2H), and *Ruminococcus bicirculans* (2I, 2J) are shown.

Among all the strains tested, *B. vulgatus* was the most sensitive to the effects of CE, with its growth being completely suppressed at 50 µg/mL. Differently, the other tested strains belonging to the genus *Bacteroides*, namely *B. eggerthii*,* B. thetaiotaomicron*, and *B. caccae*, were only slightly or not at all affected by an incubation with the maximum concentration of the CE (Figs. 1, 2a). Treatments of *B. thetaiotaomicron* and *B. caccae* with 5 and 0.5 µg/mL of CE led to an increase of the AUC values. Although no complete inhibition of the growth was detected after treatment of *A. finegoldii* (in-house isolated strain) with any of the CE concentrations tested, its growth was negatively affected by treatment with all CE concentrations. The growth of *Akkermansia muciniphila* and *Parabacteroides distasonis* (in-house isolated strain) was only slightly affected by treatment with the highest CE concentration, while *E. coli* was found to be the least sensitive Gram-negative strain to the *Alternaria* extract, as none of the incubations resulted in a change of growth kinetics. With regard to the growth of Gram-positive bacteria, none of the strains was affected by a CE-exposure to 0.5 and 5 µg/mL. Except for the type-strain of *Bifidobacterium longum*, all Gram-positive strains were affected at 50 µg/mL CE. A reduction of AUC values by 16 ± 0.7% and 16 ± 3.8% was also detected after treatment of *L. hominis* and *R. bicirculans* (in-house isolated strain) with 25 µg/mL of CE. As shown in Online Resource 3, most of the detected AUC reductions were a consequence of the reduction of the stationary-phase growth yield, without any modification of the length of the lag phase. However, a reduction of the AUC caused by an extension of the lag phase together with a decreased growth yield was instead detected after treatment of *R. bicirculans* with the highest concentration of CE tested (Fig. 2i). Of note, exposure of *A. timonensis*, *L. hominis*, and *Bifidobacterium* sp. to 50 µg/mL of CE, as well as of *A. finegoldii* to 50 µg/mL, 25 µg/mL and 5 µg/mL of CE resulted in increased values of doubling time (Online Resource 4).

#### Influence of *Alternaria* extract on biofilm production

**Fig. 3:**
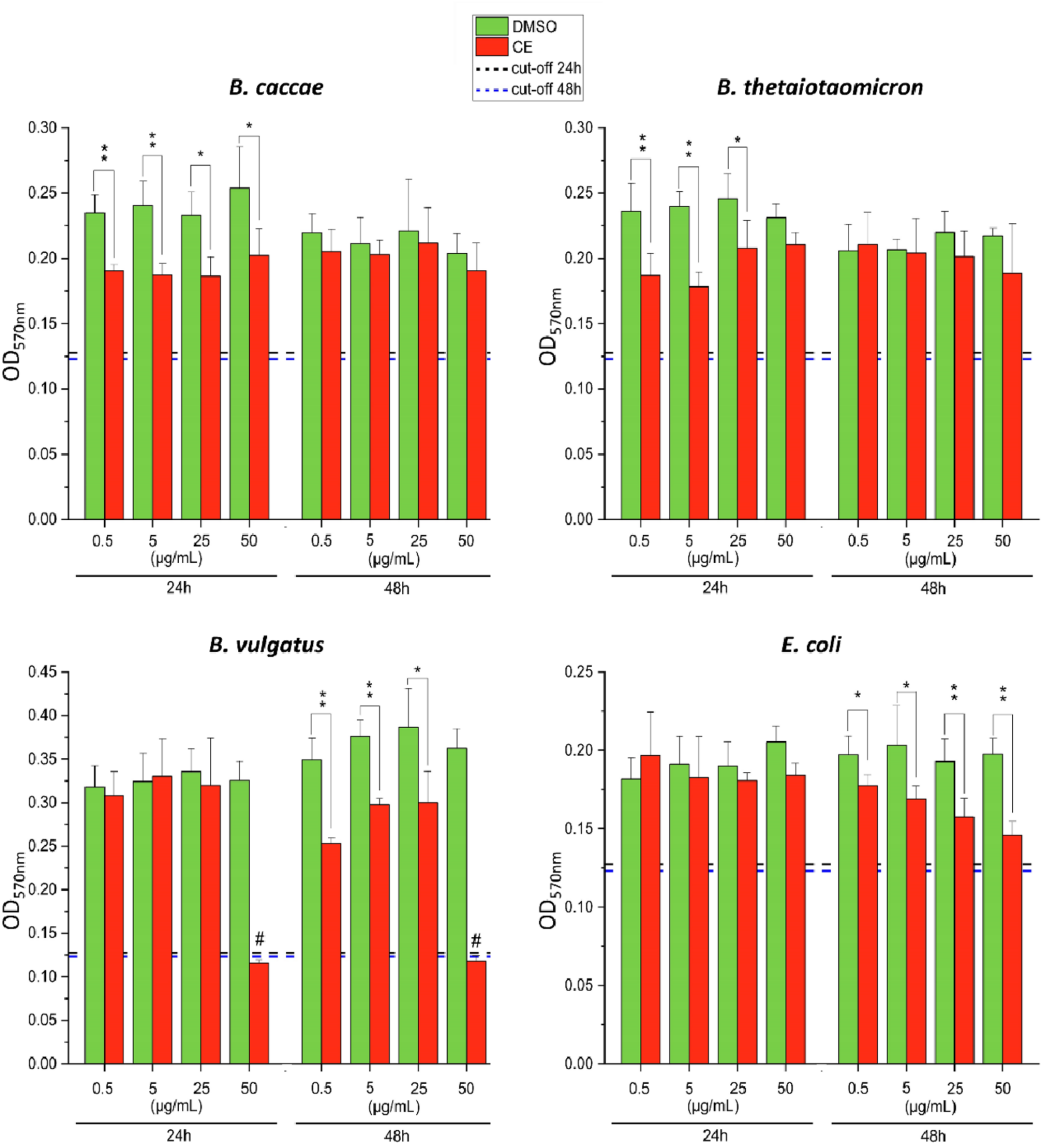
Effects on biofilm formation induced by 24 h and 48 h incubation of strains with different concentrations of the *Alternaria* extract. Differences between treated and control samples were evaluated by Student’s *t* test (*p < 0.05; **p < 0.01). Cut-off values were calculated as follow: OD_cut-off_ = mean ODs of uninoculated medium + 3 times standard deviation. ^#^Indicates no growth

The ability of the extract to affect biofilm production of gut bacteria was evaluated by employing the strains *E. coli, B. vulgatus, B. thetaiotaomicron,* and *B. caccae*. Except for the treatment of *B. vulgatus* with the maximum concentration of CE tested, all observed modifications in biofilm biomasses were attributed to the direct inhibition of biofilm formation by the CE, and not to a reduction of general bacterial growth, as confirmed through the analysis of the growth curves and the measurement of the OD_600_ values during each biofilm assay (Online Resource 5). As shown in Fig. 3, a concentration-dependent reduction of the biofilm biomass produced by *E. coli* was detected only after 48 h incubation of the strain with all CE concentrations (maximum reduction of 59 ± 10% for treatments with 50 µg/mL). Similar results were obtained for *B. vulgatus*, whose biofilm-forming capacity was not affected by 24 h incubation with the various CE concentrations, except for the treatment with the highest concentration. However, the absence of biofilm mass at this concentration cannot be traced back to a direct effect of the extract on the biofilm-forming capacity of the strain but to a growth inhibition, as previously reported and showed in Online Resource 5. After 48 h incubation, treatments of *B. vulgatus* with 25 µg/mL, 5 µg/mL, and 0.5 µg/mL led to reductions of the biofilm biomasses. The highest level of reduction (40 ± 2%) was found for the treatment with the lowest CE concentration. Exposure of *B. thetaiotaomicron* and *B. caccae* to the various concentrations of CE led to reductions of the biofilm biomasses after 24 h. However, these effects were not persistent, as no difference between treated and control samples was found after 48 h incubation. Interestingly, treatment of *B. thetaiotaomicron* with the highest CE concentration did not result in any modification of biofilm production, while the treatment with 5 µg/mL led to the most pronounced reduction in biofilm biomass (48 ± 8%). Similar results were obtained for *B. caccae*, whose strongest biomass reduction was reached after treatment with 5 µg/mL of CE (24 h). However, treatment with 50 µg/mL of CE resulted in a reduction of the biofilm biomass of 36 ± 14%.

### Effects of bacterial strains on *Alternaria* mycotoxins

#### Total recovery of *Alternaria* mycotoxins

**Fig. 4:**
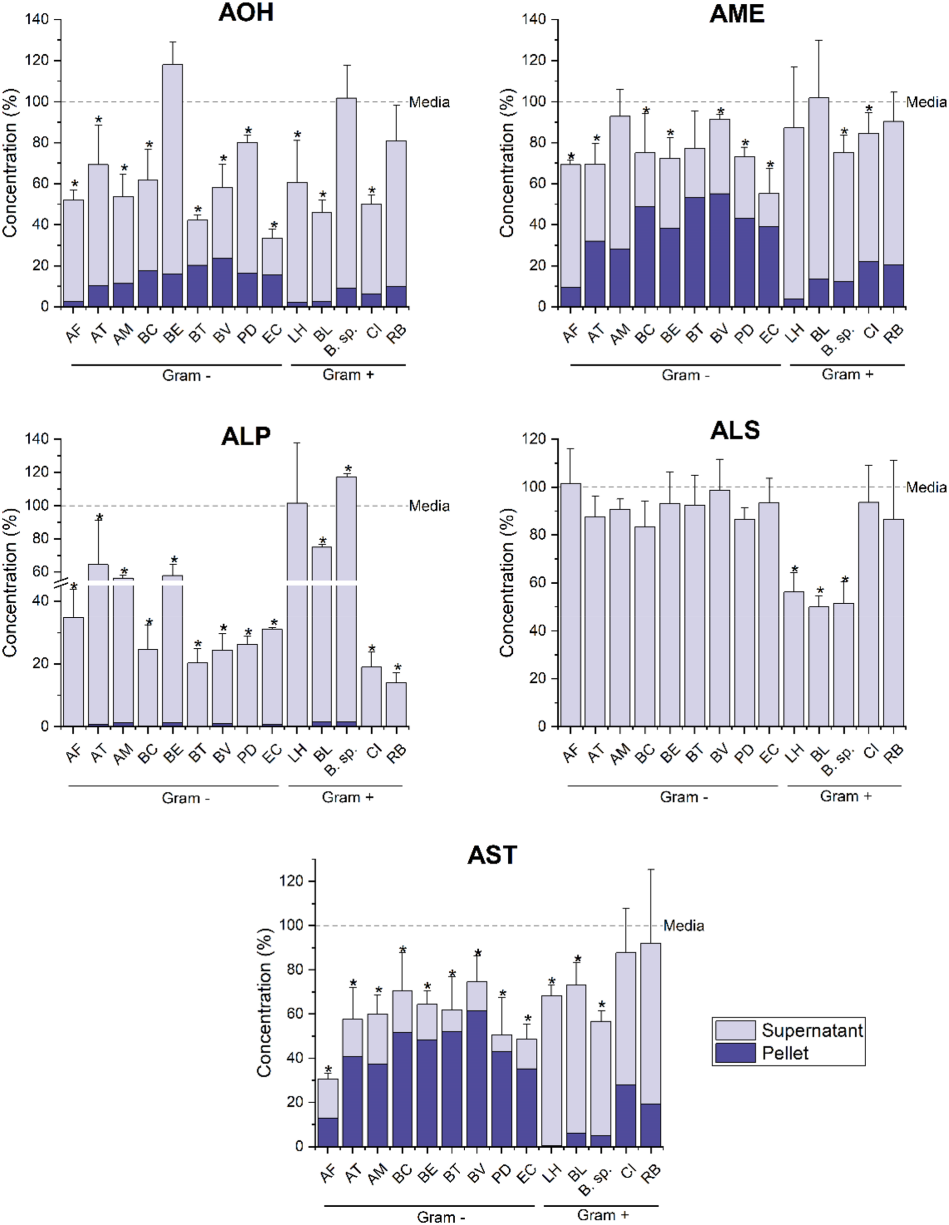
Bar charts showing the amount of the most affected mycotoxins recovered in pellets and supernatants of the tested strains after 24 h incubation with 25 µg/mL of the *Alternaria* extract. Data are reported as mean ± SD and differences between the total mycotoxin recovery in samples and media controls (media + CE) were evaluated by Student’s *t* test (*p < 0.05). *AF* *A. finegoldii*, *AT* *A. timonensis*, *AM* *A. muciniphila*, *BC* *B. caccae*, *BE* *B. eggerthii*, *BT* *B. thetaiotaomicron*, *BV* *B. vulgatus*, *PD* *P. distasonis*, *EC* *E. coli*, *LH* *L. hominis*, *BL* *B. longum*, *B. sp.* *Bifidobacterium sp.*, *CI* *C. innocuum*, *RB* *R. bicirculans*

Quantification of mycotoxins in bacterial suspensions treated with 25 µg/mL of CE, carried out by liquid chromatography tandem–mass spectrometry (LC–MS/MS), revealed that among the mycotoxins originally present in the extract, the highly reactive epoxide-carrying *Alternaria* toxins STTX-III and ATX-II, were found neither in bacteria-containing samples nor in the control media after 24 h. For this reason, they were excluded from the evaluation of the effects induced by the bacterial strains tested on the mycotoxins of the extract. The *Alternaria* mycotoxins AOH, AME, ALP, ALS, and AST were found to be the most affected ones in terms of total recovery (Fig. 4; Online Resource 6). Significant losses of AOH (*p* < 0.05), ranging from 20 ± 3% to 67 ± 4% (*P. distasonis* and *E. coli*, respectively), occurred with all tested strains, except for *B. eggerthii, Bifidobacterium sp.* (in-house isolated strain) and *R. bicirculans*. The recovery of AME, which chemically differs from AOH by an additional methyl group, was slightly higher. As for the mycotoxin ALP, the highest levels of recovery were obtained for samples incubated with the lactic acid bacteria (LAB) *L. hominis, B. longum* and *Bifidobacterium sp.*, while very low levels of recovery were obtained for the other strains. Interestingly, samples containing the strain of *Bifidobacterium sp.* were characterized by higher amounts of ALP (+ 17 ± 2%) compared to the control medium. An opposite trend was found in the total recovery of the *Alternaria* mycotoxin ALS, whose concentrations were significantly lower (*p* < 0.05) only in the LAB strains. A total recovery of AST was not obtained after incubation of the extract with most of the strains tested. ALT, TeA, TEN, and ATX-I were the mycotoxins less affected by the incubation with the various strains (Online Resources 6 and 7), since no significant difference between mycotoxin concentrations in media controls and in strain-containing samples was found for most of the strains after 24 h incubation. Notably, significant increased ATX-I concentrations (*p* < 0.05) by 42 ± 1%, 83 ± 4%, and 36 ± 6% were found in samples incubated with *B. eggerthii, P. distasonis,* and *Bifidobacterium *sp*.*, respectively.

#### Distribution of mycotoxins in pellets and supernatants of the tested strains and influence of mycotoxins lipophilicity

**Fig. 5:**
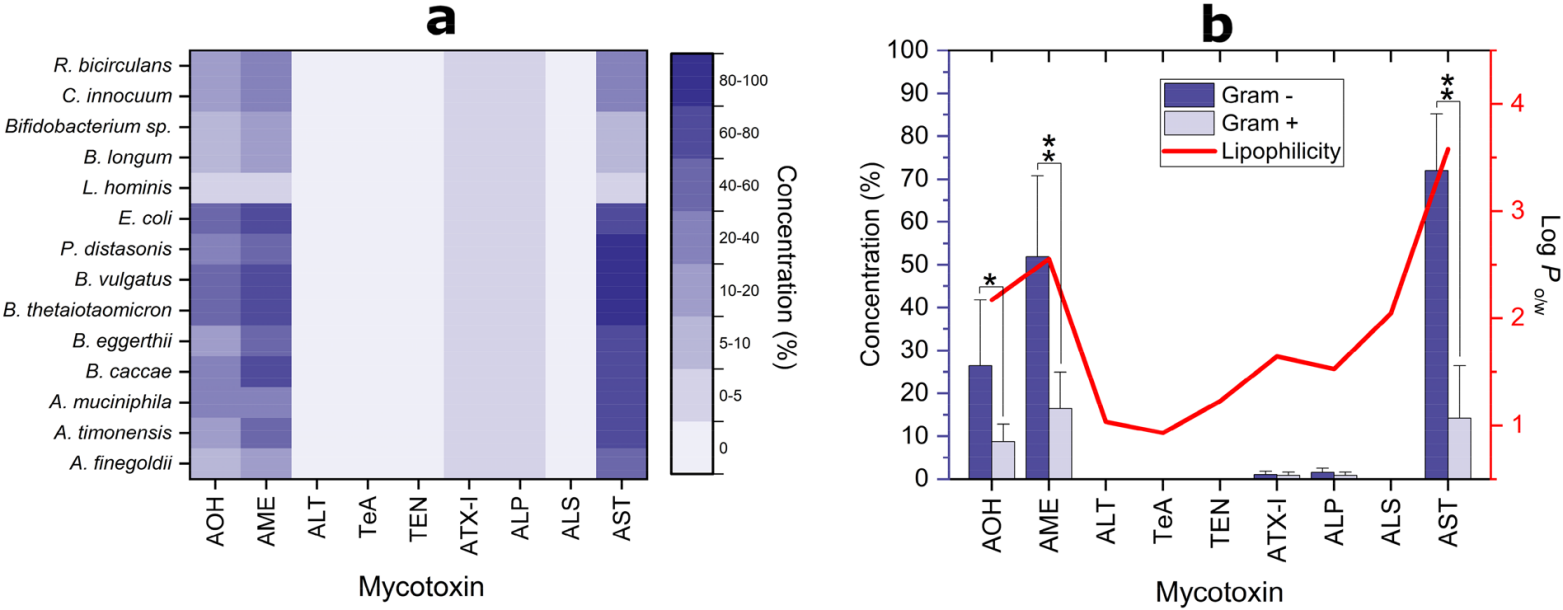
Recoveries of mycotoxins from bacterial pellets. **a** Heatmap showing the average amount (in % compared to the total amount recovered) of mycotoxins found after 24 h incubation with 25 µg/mL of CE in pellets of all strains tested. **b** Double-axis plot showing mean ± SD (in % compared to the total amount recovered) of mycotoxin concentrations found in pellets of Gram-negative and positive strains (blue and light blue columns, respectively; left axis), and the mean value of theoretical lipophilicity (solid line; right axis) of mycotoxins. Significant differences between Gram-negative and -positive strains were evaluated by Student’s *t *test (*p < 0.05; **p < 0.01)

**Fig. 6:**
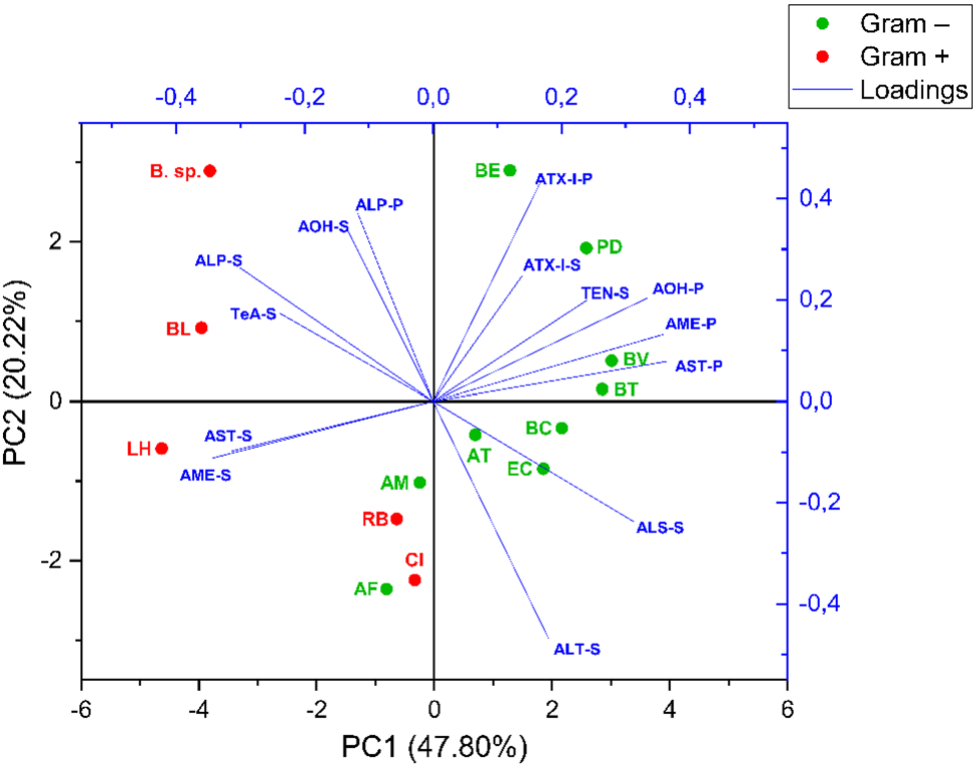
Score-loading plot of the first two principal components from PCA highlighting strain distribution in the bidimensional space and the contribution of mycotoxin concentrations found in pellets and supernatants. *AF* *A. finegoldii*, *AT* *A. timonensis*, *AM* *A. muciniphila*, *BC* *B. caccae*, *BE* *B. eggerthii*, *BT* *B. thetaiotaomicron*, *BV* *B. vulgatus*, *PD* *P. distasonis*, *EC* *E. coli*, *LH* *L. hominis*, *BL* *B. longum*, *B. sp*. *Bifidobacterium sp.*, *CI* *C. innocuum*, *RB* *R. bicirculans*

LC–MS/MS analysis of the mycotoxin concentrations found in bacterial pellets and supernatants showed a different tendency of mycotoxins to bind to bacterial cells or to remain in solution (Fig. 4; Online Resource 7). Figure 5a shows the average amount of mycotoxins found in pellets of all strains tested after 24 h incubation with 25 µg/mL of CE compared to the total amount recovered (pellet + supernatant). The mycotoxins ALT, TeA, TEN, and ALS were only found in supernatants of the tested strains, while the other mycotoxins were also found in bacterial pellets. The perylene quinone ATX-I and the mycotoxin ALP were present only at very low concentrations in pellets of the bacterial strains tested and no difference between their concentrations in pellets of Gram-positive and Gram-negative strains was observed. Among the *Alternaria* mycotoxins of the extract, a rather marked tendency of adsorption of AOH, AME, and AST by the various strains was observed (Fig. 5a). The mean concentrations of AOH found in pellets of Gram-negative strains were significantly higher (26 ± 15%; *p* < 0.05) than in pellets of Gram-positive strains (8.7 ± 4.1%), as shown in Fig. 5b. Similarly, AME concentrations in pellets of Gram-negative bacteria (52 ± 19%) were significantly different (*p* < 0.01) from those found in Gram-positive strains (16 ± 9%). The *Alternaria* mycotoxin showing the highest level of accumulation in bacterial pellets was found to be AST, whose mean recovery levels from pellets were higher (*p* < 0.01) for Gram-negative (72 ± 13%) than for Gram-positive bacteria (14 ± 12%). To avoid a possible misinterpretation of data related to mycotoxin concentrations in bacterial pellets due to the different OD600 values characterizing the bacterial suspensions, the experimentally determined mycotoxin concentrations were also normalized based on a theoretical OD600 value of 0.5 (Online Resource 8). As shown, data confirmed the tendency of mycotoxins to accumulate preferentially in the pellets of Gram-negative bacteria. The accumulation of AOH, AME, and AST within the Gram-negative bacterial pellets was a key factor contributing to the separation of Gram-positive from Gram-negative strains along the principal component (PC) 1 in the principal component analysis (PCA, Fig. 6). Gram-negative and Gram-positive bacteria distributed along the positive and negative axes of the PC1, respectively. However, four bacterial strains (*A. finegoldii*, *A. muciniphila, R. bicirculans, C. innocuum*) were overlapped and distributed along the negative axis of the PC2, thus showing mycotoxin distribution patterns similar to both Gram staining types. An in-depth investigation on the lipophilic properties of the *Alternaria* mycotoxins contained in the extract, performed using the SwissADME online tool, revealed a direct correlation between the presence of mycotoxins in pellets and the in silico predicted values of lipophilicity of mycotoxins (Fig. 5b). The highest value for the n-octanol/water partition coefficient (log P_o/w_) was obtained for AST (3.58 ± 1.24), followed by AME (2.55 ± 0.62), AOH (2.17 ± 0.60), ALS (2.05 ± 0.65), ATX-I (1.64 ± 0.77), and ALP (1.53 ± 0.66). Although these results are in accordance with the mycotoxin concentrations found in pellets, ALP was not found in any pellet of the tested strains. Of note, the mycotoxins not found in the pellets of the tested strains (i.e. ALT, TeA, and TEN) were characterized by the lowest log P_o/w_ predicted levels (1.03 ± 0.51, 0.93 ± 0.76, and 1.23 ± 1.36, respectively).

## Discussion

A preliminary investigation examining the potential of the *Alternaria* extract to completely inhibit the growth of five selected strains (*B. thetaiotaomicron, B. vulgatus, B. caccae, C. innocuum* and *E. coli*) revealed no or low activity of the extract. Considerable MIC values were found only for *B. vulgatus* (50 µg/mL) and *C. innocuum* (100 µg/mL), while the other strains were not affected even at the maximum concentration of the CE (200 µg/mL). Despite this, most of the bacteria exhibited changes in their growth kinetics after exposure to specific concentrations of the extract (Fig. 1; Online Resource 3). A representative set of examples is reported in Fig. 2. In particular, while the AUC of *A. finegoldii* was reduced by the exposure to both maximum (Fig. 2a) and minimum (Fig. 2b) concentrations of CE, no changes in growth kinetics were found for *E. coli* (Fig. 2g, h) and the ATCC 15,697 strain of *B. longum* (Fig. 1), which were the most resistant bacteria against *Alternaria* toxins in study at hand. Strains belonging to the genus *Bacteroides* deserve particular attention because of the high intra-genus variability in their response to the presence of the *Alternaria* extract. Exposure of *B. vulgatus* to the maximum CE concentration resulted in growth inhibition (Fig. 2e), while exposure to the lowest CE concentration did not affect its growth (Fig. 2f). Exposure of *B. eggerthii* and *B. thetaiotaomicron* to the maximum CE concentration only slightly affected their growth, while no changes were found for *B. caccae* at this concentration*.* The strains *B. thetaiotaomicron* and *B. caccae* exhibited increased AUC values after exposure to the lowest CE concentrations tested (5 µg/mL and 0.5 µg/mL). This might be the consequence of a mycotoxin-induced modulation of intra-cellular pathways involved in bacterial growth, or the result of a possible utilization of some of the *Alternaria* mycotoxins as carbon and energy sources. Supporting this interpretation, bacteria were previously reported to metabolize xenobiotics, and the demethylation of compounds can provide a carbon source for their growth (Koppel et al. 2017). However, considering the lack of data in the literature, further studies are required to better clarify this phenomenon. All the observed modifications in bacterial growth discussed above were found to be mainly attributable to modifications of the stationary-phase growth yield and, in some cases, increased doubling times were also observed (Online Resource 4), suggesting a manifold action on bacterial growth worth of being further analyzed. *R. bicirculans* was the only strain for which an extended duration of the lag phase could be observed after treatment with 50 µg/mL of CE (Fig. 2i). The prolongation of the lag phase can be considered a defense mechanism that allows bacteria to survive unfavorable growth conditions, such as presence of antimicrobial compounds. As an example, the growth of a clinical isolate of *Enterococcus faecium* was delayed by the treatment with the fluoroquinolones ciprofloxacin and moxifloxacin, which act by inhibiting the DNA gyrase (Theophel et al. 2014). Interestingly, some of the *Alternaria* mycotoxins contained in the extract (i.e. AOH, AME, ATX-I, ATX-II, ALP, and STTX-III) were previously reported to target the bacterial gyrase (Jarolim et al. 2017). Consistently with the growth inhibitions and changes in the growth kinetics observed in the present study, the ability of *Alternaria* mycotoxins to exert antimicrobial effects was previously reported by several authors. AOH inhibited the growth of *Staphylococcus aureus* and *Corynebacterium betae* at micromolar concentrations, while the growth of an *E. coli* strain was only slightly reduced (Freeman 1966). AME and ALT were active against both Gram-positive and Gram-negative bacteria (Pero et al. 1971; Lou et al. 2016). The mycotoxin ALS exhibited antimicrobial activity against several multidrug-resistant bacteria (Kjer et al. 2009), while AST only inhibited the growth of pathogenic Gram-positive strains (MIC values ranging from 0.12 to 2 µg/mL) (Hellwig et al. 2002). The concentrations of AST the strains tested in the present study were exposed to (see Online Resource 9) were in the MIC range reported above (Hellwig et al. 2002). Hence, this mycotoxin might have at least contributed not only to the changes of the growth kinetics of the Gram-positive bacteria, but also to the growth inhibition of *C. innocuum* (Gram-positive) after treatment with 100 µg/mL of CE. However, considering that the other *Alternaria* mycotoxins of the extract were present in significantly lower concentrations (Online Resource 9) than those previously reported in literature to be able to exert antimicrobial effects (Freeman 1966; Pero et al. 1971; Kjer et al. 2009; Lou et al. 2016) and that the growth of Gram-negative bacteria was also influenced, the observed effects might be the consequence of synergistic effects caused by the co-presence of multiple *Alternaria* mycotoxins.

It is important to note that some of the mycotoxin concentrations tested could be reached in the GI tract upon consumption of contaminated food. In fact, assuming a complete bio-accessibility of mycotoxins, their dilution in a total volume of gastrointestinal fluids of 1.1 L (maximum volume of gastrointestinal fluids reported by Schiller et al. 2005), an intake of 315 g of sweet peppers, according to the maximum concentrations of mycotoxins found by Gambacorta et al. (2018), would be sufficient to exceed the concentrations of AOH and ALT tested in the present study. As for AME, TEN and TeA, less than 50 g of soya beans, 50 g of bread and 250 g of tomatoes would be sufficient according to the contamination reported by Oviedo et al. (2012), Zhao et al. (2015) and Stinson et al. (1981), respectively. Unfortunately, the shortage of occurrence and exposure data related to the other mycotoxins of the extract prevents a precise calculation. Anyway, based on this and the results obtained in the present work, further studies are necessary to clarify the actual occurrence of these mycotoxins in food on the one hand, and in vivo investigations aimed at evaluating the effects of *Alternaria* mycotoxins on the gut microbiome would be desirable on the other hand, considering the current lack of data and the possible negative consequences resulting from changes of its composition. Indeed, gut dysbiosis, characterized by a reduced or increased presence of specific bacteria or groups of bacteria, has been linked both to the development of pathologies related to digestive tract and other organ systems (Carding et al. 2015). As an example, a possible involvement of some pathobiont strains of *E. coli* in the pathogenesis of inflammatory bowel disease (IBD), which includes Crohn's disease (CD) and ulcerative colitis (UC), has been suggested (Perna et al. 2020). Patients with CD were found to have a higher abundance of *E. coli* at the level of the ileum than healthy people (Perna et al. 2020). On the contrary, the abundance of *A. muciniphila*, which is one of the most important mucolytic symbionts inhabiting the human intestine, was found to be reduced in IBD, autism, and obesity (Derrien et al. 2017). A lower abundance of the genus *Bacteroides*, compared to healthy controls, was also reported in CD and UC patients in active phase (Zhou and Zhi 2016).

Although it has not yet been clarified whether these alterations in the abundance of intestinal bacteria are a cause or a consequence of IBD, the modifications in bacterial growth observed in the present work raise the question whether exposure to *Alternaria* toxins may actually represent a contributing factor to the development of diseases, such as IBD.

An in-depth investigation with selected strains (*E. coli, B. vulgatus, B. thetaiotaomicron* and *B. caccae*) revealed also the ability of the *Alternaria* extract to affect biofilm formation. As already known, microorganisms inhabiting the human intestinal tract live as complex biofilm communities in close association with the outer layer of the host mucus (Buret et al. 2019). The disruption of the complex structure of the biofilm, which confers microorganisms increased tolerance to stress, may lead not only to diseases at the gastrointestinal level, but also in other organs (Buret et al. 2019). In the present study, the biofilm production by *E. coli* was reduced in a concentration-dependent manner after 48 h incubation with all concentrations of CE, without changes in the growth of the strain. Similar results were reported by Lee et al. (2014), who reported the ability of coumarin and eight derivatives to inhibit the formation of biofilms produced by a pathogenic *E. coli* strain. Of note, treatment with 50 µg/mL of ellagic acid (derivative), which is structurally related to some of the *Alternaria* mycotoxins (AOH, AME), led to the reduction of about 40% of the biofilm biomass produced by the strain (Lee et al. 2014). Although ellagic acid was not tested, coumarin and the two derivatives umbelliferone and esculetin were found to repress curli genes and motility genes. In addition, the observed reductions in fimbriae production and swarming motility were linked to the observed transcriptional modifications. On this basis, it cannot be excluded that, in the present study, some of the *Alternaria* mycotoxins might have triggered changes in the transcription of genes involved in biofilm formation. Unlike *E. coli*, the reduction in biofilm biomass observed for strains belonging to the genus *Bacteroides* did not follow a clear concentration-dependent pattern. In addition, while the biofilm production of *B. vulgatus* was affected after 48 h incubation, reduction of biomasses was found for *B. caccae and B. thetaiotaomicron* after 24 h, but not after 48 h. This suggests possible losses of the antibiofilm properties of the extract over time or the onset of adaptive cellular responses to mycotoxin-induced stress. The detailed elucidation of underlying mechanisms will be addressed in subsequent studies. Apart from the differences observed in the time of onset of inhibitory effects on biofilm production (i.e. 24 h or 48 h), one of the most interesting results obtained from the analysis of data related to the production of biofilms was the most effective inhibition of biofilm formation observed in strains of the genus *Bacteroides* exposed to the lowest CE concentrations. Interestingly, similar results have been reported upon treatment of bacterial strains with both pure compounds (i.e. antibiotics; Majidpour et al. 2017) and complex mixtures (i.e. essential oils; Papa et al. 2020). Despite no information is currently available about both the mechanisms underlying this phenomenon and the anti- or pro-biofilm properties of the single compounds of the *Alternaria* extract, it could be hypothesized that some mycotoxins of the extract exert pro-biofilm effects, while others act as anti-biofilm factors, resulting, in mixture, in an inhibition of the biofilm production. However, at the lowest CE concentrations, the pro-biofilm mycotoxins may not be within their concentration range of biological activity to exert effects in the strains of the genus *Bacteroides*, thus they might not be able to counteract the anti-biofilm effects of the other mycotoxins of the extract.

To evaluate the ability of the tested strains to chemically modify *Alternaria* toxins, quantification of mycotoxins in bacterial pellets and supernatants was performed by LC–MS/MS analysis. Results clearly showed no or low impact of bacterial strains on the total recovery of ALT, TeA, TEN, and ATX-I (Online Resource 7). An increased concentration of ATX-I after 24 h incubation was found in samples incubated with *B. eggerthii*,* P. distasonis*, and *Bifidobacterium sp.* compared to the controls without bacteria. These higher concentrations might be a consequence of de-epoxidation processes involving the highly reactive epoxide-carrying mycotoxin ATX-II. In fact, an in vivo study carried out by Puntscher et al. (2019a) reported the presence of ATX-I in plasma, urine and fecal samples of rats administered with the *Alternaria* mycotoxin ATX-II. This transformation was also reported in eukaryotic cell lines (Fleck et al. 2014a, b). Moreover, in a recent study (Crudo et al. 2020), we found higher concentrations of ATX-I in samples of fecal slurries of 3 out 4 donors after a 3 h anaerobic incubation with the same *Alternaria* extract used in the present study. The ability of gut microorganisms from broilers, pigs, and rats to transform the epoxide-carrying compounds DON into de-epoxy-DON was also reported (Worrell et al. 1989; He et al. 1992; Kollarczik et al. 1994; Young et al. 2007). Although in the present study, ATX-II was found neither in samples containing bacteria nor in media controls after 24 h incubation, in our previous study, we reported ATX-II to be still present after 3 h incubation (Crudo et al. 2020). Thus, it is probable that the transformation of ATX-II into ATX-I mediated by *B. eggerthii, P. distasonis*, and *Bifidobacterium *sp*.* occurred in the first hours after incubation with the *Alternaria* extract.

The transformation of the epoxide-carrying *Alternaria* mycotoxin STTX-III into ALP was also reported in literature (Fleck et al. 2014a, b). In the present study, most of the bacteria mediated a reduction of the total content of ALP. Of note, ALP concentrations in samples incubated with *Bifidobacterium sp.* were higher than those present in the control media, suggesting a possible transformation of STTX-III to ALP by this bacterium. Among the other *Alternaria* mycotoxins, significant unrecovered amounts of AOH, AME, and AST were found in samples incubated with most of the strains, without any difference between Gram staining types. The incomplete recovery of AOH was also reported by Lemke et al. (2016) who recovered percentages of the mycotoxin ranging from 70 to 85% from pure cultures of *E. coli DH5α* and *L. plantarum* BFE5092. However, no data are currently available about the total recovery of the other mycotoxins after incubation with pure cultures, including ALS, for which we observed an incomplete recovery in samples from the three strains of LAB. The uncomplete recovery obtained for some mycotoxins might be a consequence of their metabolization by the bacterial strains tested. As a matter of fact, the gut microbiota has been already reported to modify the chemical structure of several compounds, including food components and contaminants (Koppel et al. 2017). To date, although no information is available about gut microbial metabolites of the *Alternaria* mycotoxins, several polyphenolic compounds, having chemical structures similar to some *Alternaria* mycotoxins, have been reported to be metabolized by the gut microbiome (Makarewicz et al. 2021). Considering that the profile of metabolites produced depends not only on the chemical structure of the original compound but also on the repertoire of enzymes expressed in the intestinal microbiome, which can vary on an individual basis (Koppel et al. 2017), it is difficult to hypothesize the chemical structure of possible metabolites of *Alternaria* mycotoxins. However, the mycotoxin AOH has a chemical structure very similar to urolithins, a class of gut microbial metabolites produced from ellagic acid and, among which, urolithin C has recently been reported to reduce the absorption and metabolism of AOH in a Caco-2 cell model exploiting its chemical analogies to AOH (Crudo et al. 2021). Of note, the production of the various urolithins occurs through successive removal of hydroxyl groups (Espín et al. 2013). Based on this, a loss of hydroxyl groups might not only occur for the mycotoxin AOH, but also for AME, which differs from AOH by an additional methyl group (Online Resource 1). In addition, with regard to the mycotoxin AST, the exocyclic diene residue present in its structure could be a possible target for gut microbial metabolism, considering the well-known ability of the gut microbiome to reduce several functional groups, including alkenes (Koppel et al. 2017).

Anyway, as shown in Figs. 4, 5, LC–MS/MS data analysis revealed the tendency of AOH, AME, and AST to accumulate within bacterial pellets. Albeit to a much lesser extent, this phenomenon was also observed for ATX-I and ALP, while ALT, TeA, TEN, and ALS were only found in supernatants. This is in line with Król et al. (2018) who reported the ability of bacteria to bind mycotoxins.

Several parameters are considered to affect the ability of compounds to penetrate the bacterial cell walls (Macielag 2012). Among these, the log P_o/w_ value, which is a measure of lipophilicity of molecules, plays an important role because compounds having high hydrophilicity are excluded from the passive passage through the lipid bilayer (Fost and John 1997; Macielag 2012). In the present work, a direct relation between mycotoxin concentrations in pellets and their theoretical log P values was observed (Fig. 5b). The accumulation of mycotoxins in or on bacterial cells was most pronounced for AST (up to 84.9% in relation to the total recovered amount, in *P. distasonis*; log *P* = 3.58 ± 1.24), followed by AME (70.57%, *E.coli*; log *P* = 2.55 ± 0.62), and AOH (47.91%, *B. eggerthii*; log *P* = 2.17 ± 0.60). The mycotoxins ATX-I and ALP accumulated to considerably less amounts, which was accompanied with low log *P* values (1.64 ± 0.77 and 1.53 ± 0.66, respectively). The theoretical log *P* values of the other *Alternaria* mycotoxins not found in bacterial pellets were all below these values, except for ALS whose log *P* was between those of AOH and ATX-I (Fig. 5b).

Although in the present study, both Gram staining types were able to adsorb AOH, AME, and AST, Gram-negative bacteria showed higher adsorptive capacities (Fig. 5b). This tendency was not observed for ATX-I and ALP, which were present in small comparable amounts within both Gram-negative and Gram-positive bacteria. Of note, the preferential accumulation of AOH, AME, and AST into Gram-negative bacterial pellets was also observed after normalization of data based on a theoretical OD600 value of 0.5 (Online Resource 8). However, differences in terms of mycotoxin concentrations in pellets between the experimental and normalized data were observed and, in some cases, data normalization resulted in apparently inconsistent results such as apparent accumulation levels exceeding 100%. This can be attributed to the mathematical procedure followed during the calculation which does not take into account the possible existence of a non-linear correlation between the amount of bacteria and the level of adsorbed mycotoxins due to achievement of saturation conditions or depletion of adsorbable mycotoxins. Anyway, the greater tendency of Gram-negative bacteria to accumulate AOH was also reported by Lemke et al. (2016), while no studies are currently available about the other *Alternaria* mycotoxins. The different levels of mycotoxin accumulation between the two Gram staining types might be explained by the different structure of the bacterial cell walls. Gram-negative bacteria possess a lipopolysaccharide-coated outer membrane and an inner cytoplasmic cell membrane which are separated by a thin layer of peptidoglycan. On the contrary, the cell wall surrounding Gram-positive bacteria is composed by a thick layer of peptidoglycan, but lacks the outer membrane (Denyer and Maillard 2002). Thus, the high lipid content of the Gram-negative cell wall might have favored the accumulation of the mycotoxins showing the highest log P values. However, considering that the use of LC–MS/MS analysis is not suitable to determine the exact location of compounds within bacterial cells, further studies are required to elucidate this point. Interestingly, the ability of some *Alternaria* mycotoxins (i.e. ATX-II and AOH) to interact with eukaryotic cell membranes has already been reported (Del Favero et al. 2018,2020a,b). In particular, the mycotoxin AOH, which is structurally similar to cholesterol, was found to affect the membrane fluidity of THP-1 macrophages and to intercalate in cholesterol-rich membrane domains (Del Favero et al. 2020b). Although sterols are not typically found in bacterial membranes and bacteria are unable to synthesize cholesterol (Huang and London 2016), this sterol was previously found in membranes of strains belonging to the genera *Mycoplasma*,* Ehrlichia*,* Anaplasm*,* Brachyspira*,* Helicobacter *and *Borrelia*, probably due to the ability of these strains to acquire cholesterol from cell hosts (Huang and London 2016). In addition, the bacterial hopanoids, which are structurally and functionally similar to sterols, were found to interact with glycolipids in bacterial outer membrane. All these findings support the possibility that some of the *Alternaria* mycotoxins contained in the extract were accumulated in the bacterial lipid bilayer (Sáenz et al. 2015).

## Conclusion

This is the first in vitro study investigating the bidirectional relationship between the emerging class of *Alternaria* mycotoxins and human gut bacterial strains. Results obtained show that these potential food contaminants possess the ability to affect the growth of human gut bacterial strains and their ability to produce biofilms, suggesting that they may impair the composition and activity of the complex microbial community inhabiting human intestine. Importantly, human gut-derived microbial strains were found to adsorb some *Alternaria* toxins, thus indicating an important role of the gut microbiome in modulating the toxicity of these mycotoxins in humans. Taken together, this study highlights the potential role of mycotoxins in impacting gut microbiota and, vice versa, the importance of the latter in mediating the systemic bioavailability and detoxification of these abundant food contaminants consumers are constantly exposed to. Considering the well-known relation between gut microbiota and human health, subsequent in vivo studies are required to better characterize the actual risk related to this class of food contaminants. In addition, the incomplete recovery of some *Alternaria* mycotoxins poses the question whether gut microorganisms may transform these mycotoxins into unknown metabolites, which might also retain a certain degree of toxicity. To address this question, further studies based on untargeted high-resolution MS approaches, are first of all needed to clarify the fate of these fungal metabolites.

## Supplementary Information

Below is the link to the electronic supplementary material.

Supplementary file1 (DOCX 2225 KB)

## Abbreviations

AOH: Alternariol
AME: Alternariol monomethyl ether
ALT: Altenuene
TeA: Tenuazonic acid
TEN: Tentoxin
ALP: Alterperylenol
AST: Altersetin
ATX: Altertoxin
STTX-III: Stemphyltoxin III
CE: Complex extract of *Alternaria* mycotoxins

## Declarations

### Availability of data and material

The dataset generated during the current study is available from the corresponding author on reasonable request.
