# Supplementary material for: In vitro interactions of Alternaria mycotoxins, an emerging class of food contaminants, with the gut microbiota: a bidirectional relationship
Source: Arch Toxicol. 2021 Apr 13;95(7):2533–49. doi: 10.1007/s00204-021-03043-x (PMC8241668; doi:10.1007/s00204-021-03043-x)
Supplement: Supplementary file 1 — Supplementary file1 (DOCX 2225 KB) [file 204_2021_3043_MOESM1_ESM.docx]

**Supplementary material**

***In vitro* interactions of *Alternaria* mycotoxins, an emerging class of food contaminants, with the gut microbiota: a bidirectional relationship.**

Francesco Crudo^1,2^, Georg Aichinger^1^, Jovana Mihajlovic^3^, Elisabeth Varga^1^, Luca Dellafiora^2^, Benedikt Warth^1^, Chiara Dall’Asta^2^, David Berry^1,3,4^, Doris Marko^1,2.*^

Author affiliations:

^1^ Department of Food Chemistry and Toxicology, Faculty of Chemistry, University of Vienna, Währinger Str. 38, 1090 Vienna, Austria;

^2^ Department of Food and Drug, University of Parma, Area Parco delle Scienze 27/A, 43124 Parma, Italy;

^3^ Department of Microbiology and Ecosystem Science, Centre for Microbiology and Environmental Systems Science, University of Vienna, Althanstr. 14, 1090 Vienna, Austria;

^4^ Joint Microbiome Facility of the Medical University of Vienna and the University of Vienna.

Corresponding author:

Prof. Dr. Doris Marko
Department of Food Chemistry and Toxicology, University of Vienna
Währinger Str. 38, 1090 Wien, Austria
doris.marko@univie.ac.at

**Table of Contents**

[**Online Resource 1** Chemical structures of the mycotoxins contained in the extract. 3](#_Toc63980305)

[**Online Resource 2** Bacterial strains and media employed in the study. 4](#_Toc63980306)

[**Online Resource 3** Growth curves of the strains exposed to various concentrations of the complex *Alternaria* extract (CE).. 5](#_Toc63980307)

[**Online Resource 4** Doubling time of the strains exposed to the various concentrations of the *Alternaria* extract, each in comparison to the respective solvent control (0.1-0.001% DMSO). 12](#_Toc63980308)

[**Online Resource 5.** OD_600_ values recorded after 24 h and 48 h incubation with various concentrations of the *Alternaria* extract, each in comparison to the respective solvent control (0.1-0.001% DMSO). 13](#_Toc63980309)

[**Online Resource 6.** Total recovery of *Alternaria* mycotoxins after 24 h incubation of strains with 25 µg/mL of CE 14](#_Toc63980310)

[**Online Resource 7** Bar charts showing the amount of the least affected mycotoxins recovered in pellets and supernatants of the tested strains after 24 h incubation with 25 µg/mL of CE.. 15](#_Toc63980311)

[**Online Resource 8** Theoretical recoveries of mycotoxins from bacterial pellets (normalized based on a theorethical OD600 of 0.5). 16](#_Toc63980312)

[**Online Resource 9** Concentrations of mycotoxins the bacterial strains were exposed to during treatments with various concentrations of the *Alternaria* extract. 17](#_Toc63980313)


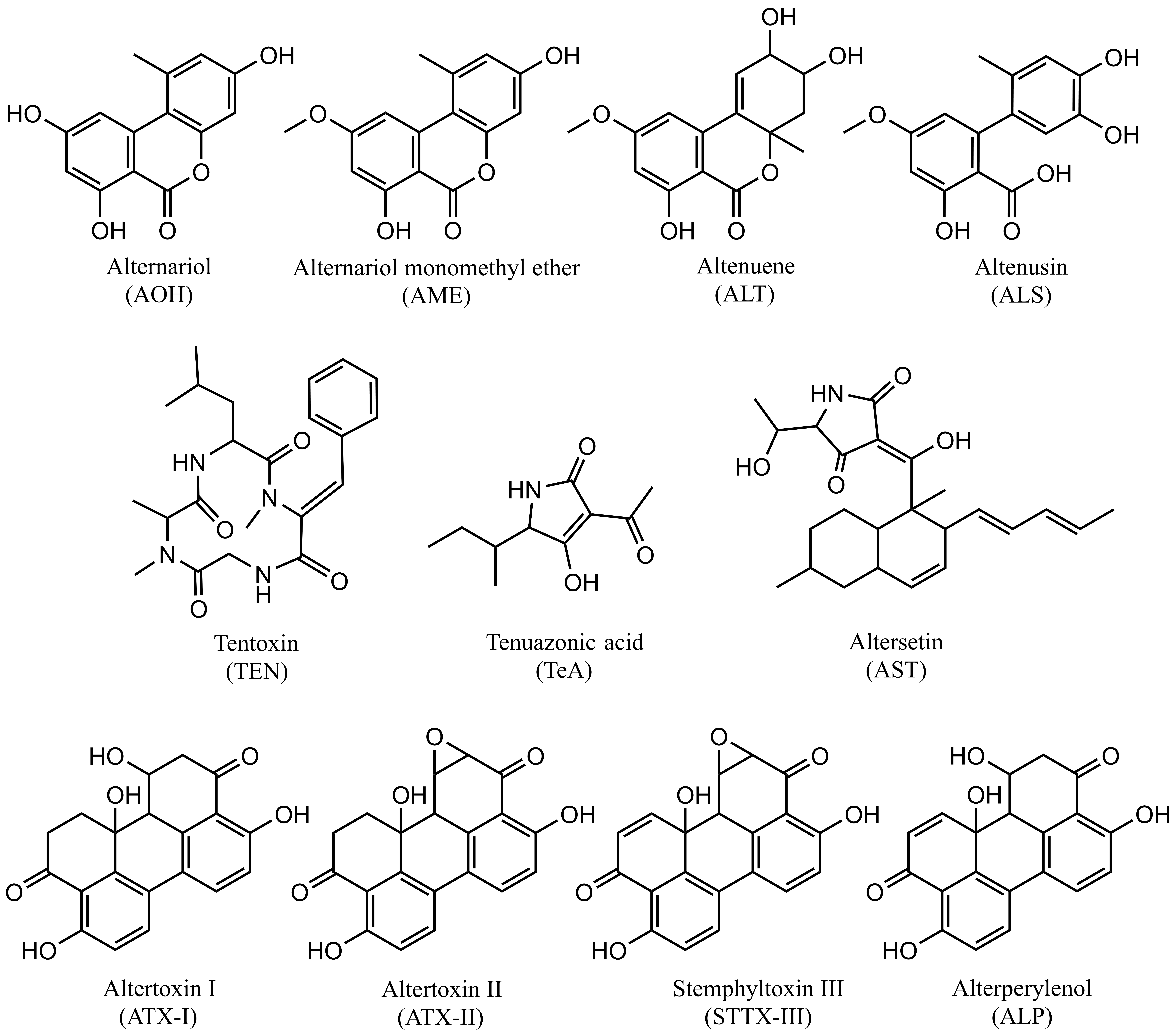


# **Online Resource 1.** Chemical structures of the *Alternaria* mycotoxins contained in the extract.

# **Online Resource 2.** Bacterial strains and media employed in the study.

| Strain^a^ | Abbreviation | Received from | Phylum | Gram staining | Medium^b^ |
| --- | --- | --- | --- | --- | --- |
| *Alistipes finegoldii_*AR1 (99% 16S rRNA gene sequence similarity with *A. finegoldii* DSM 17242) | *AF* | *Own isolation* | Bacteroidetes | – | YCFA-g |
| *Alistipes timonensis DSM25383* | *AT* | *DSMZ* | Bacteroidetes | – | YCFA-g |
| *Akkermansia muciniphila DSM22959* | *AM* | *DSMZ* | Verrucomicrobia | – | BHI-muc |
| *Bacteroides caccae DSM19204* | *BC* | *DSMZ* | Bacteroidetes | – | m-BHI |
| *Bacteroides eggerthii DSM20697* | *BE* | *DSMZ* | Bacteroidetes | – | m-BHI |
| *Bacteroides thetaiotaomicron DSM2079* | *BT* | *DSMZ* | Bacteroidetes | – | m-BHI |
| *Bacteroides vulgatus DSM1447* | *BV* | DSMZ | Bacteroidetes | – | m-BHI |
| *Parabacteroides distasonis_*AR2 (99% 16S rRNA gene sequence similarity with *P. distasonis* DSM 20701^1^) | *PD* | *Own isolation* | Bacteroidetes | – | m-BHI |
| *Escherichia coli ATCC23716* | *EC* | *ATCC* | Proteobacteria | – | m-BHI |
| *Lactobacillus hominis DSM23910* | *LH* | *DSMZ* | Firmicutes | + | m-MRS |
| *Bifidobacterium longum subsp. infantis* *ATCC 15697* | *BL* | ATCC | Actinobacteria | + | m-MRS |
| *Bifidobacterium_*sp.AR3 (95% 16S rRNA gene sequence similarity with *B. longum^1^*) | *B. sp* | *Own isolation* | Actinobacteria | + | m-MRS |
| *Clostridium innocuum DSMZ1286* | *CI* | *DSMZ* | Firmicutes | + | m-BHI |
| *Ruminococcus bicirculans _*AR4 (99% 16S rRNA gene sequence similarity with *Ruminococcus bicirculans 80/3^1^)* | *RB* | *Own isolation* | Firmicutes | + | YCFA-g |

^a^ In-house isolated strains are identified to genus or species level based on the best BLAST (Morgluis *et al.* 2008, *Bioinformatic*s; https://pubmed.ncbi.nlm.nih.gov/18567917/) result for their 16S rRNA gene sequence similarity.

^b^ YCFA-g: yeast extract-casein hydrolysate-fatty acids broth supplemented with glucose; BHI-muc: brain heart infusion broth supplemented with mucin; m-BHI: modified brain heart infusion broth; m-MRS: modified De Man, Rogosa, Sharpe broth.


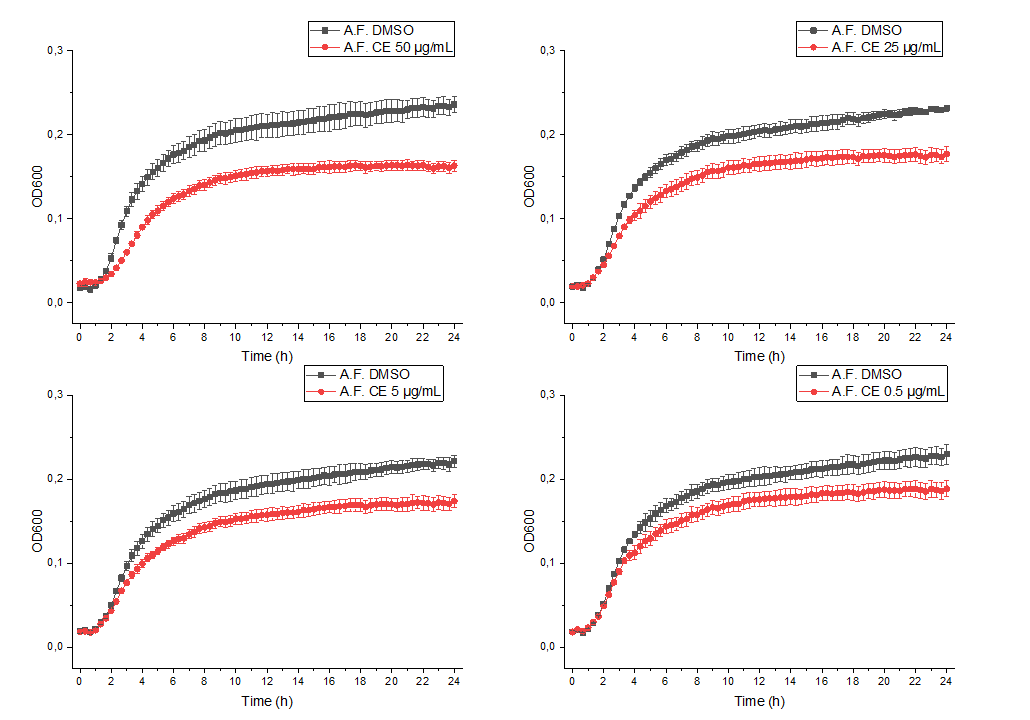


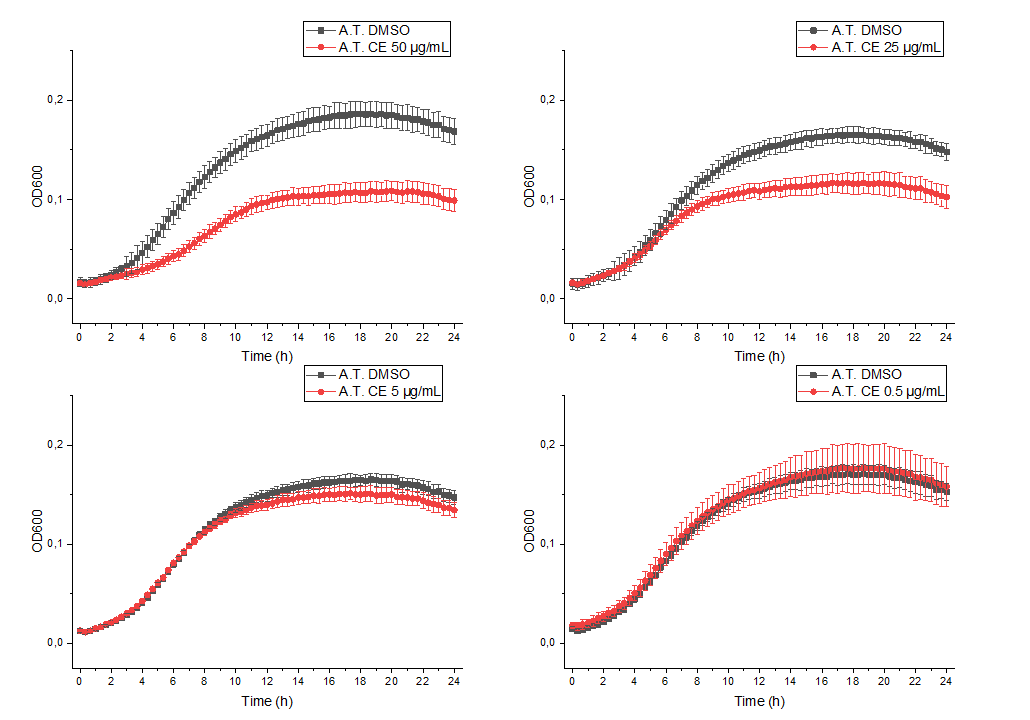


# **Online Resource 3.** Growth curves of the strains exposed to various concentrations of the complex *Alternaria* extract (CE). The growth curves are expressed as mean values (three replicates) ± SD of optical densities measured at 600 nm. DMSO indicate the growth curves of the respective controls (0.1% - 0.001% DMSO).

AF: *A. finegoldii*; AT: *A. timonensis*; AM: *A. muciniphila*; BC: *B. caccae*; BE: *B. eggerthii*; BT: *B. thetaiotaomicron*; BV: *B. vulgatus*; PD: *P. distasonis*; EC: *E. coli*; LH: *L. hominis*; BL: *B. longum*; B. sp.: *Bifidobacterium sp.*; CI: *C. innocuum*; RB: *R. bicirculans* .


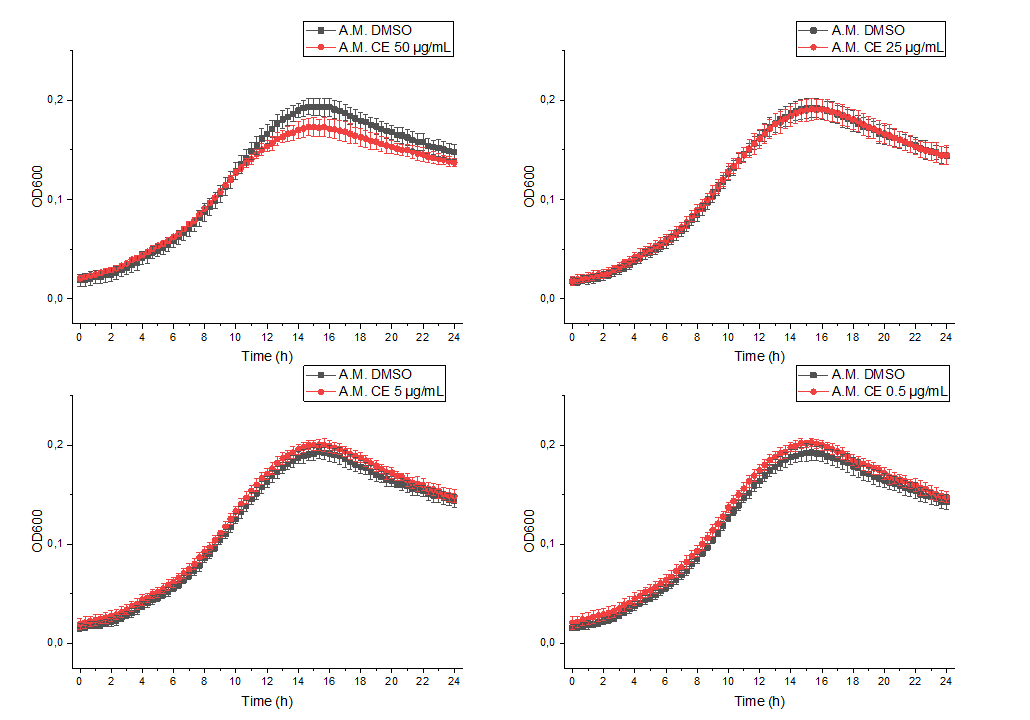


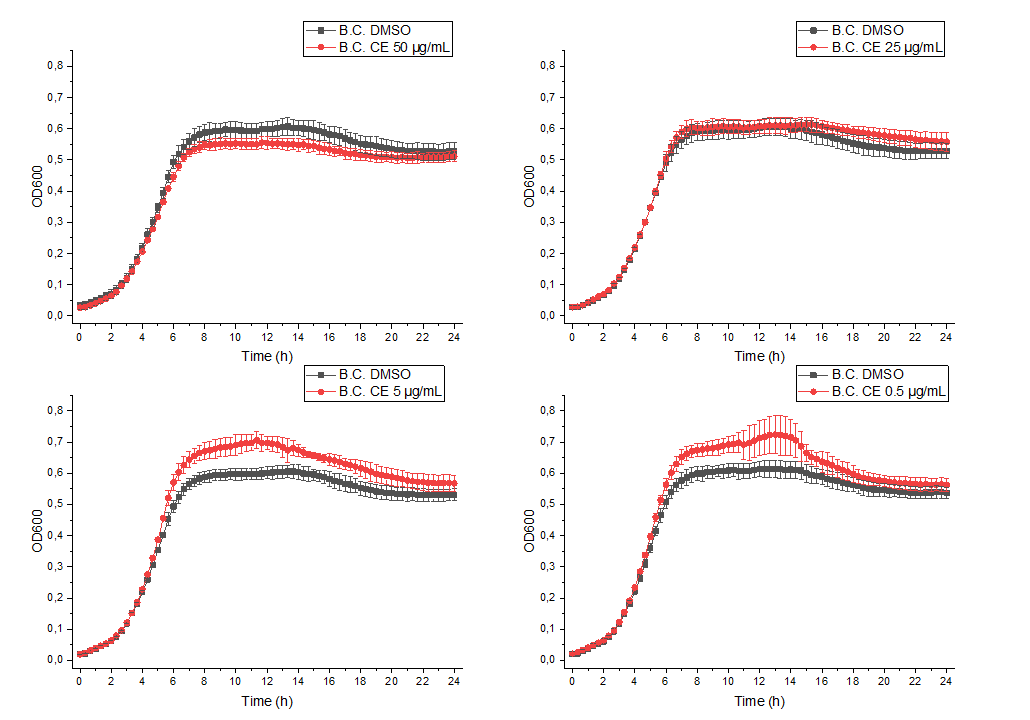


**Online Resource 3.** Continue.


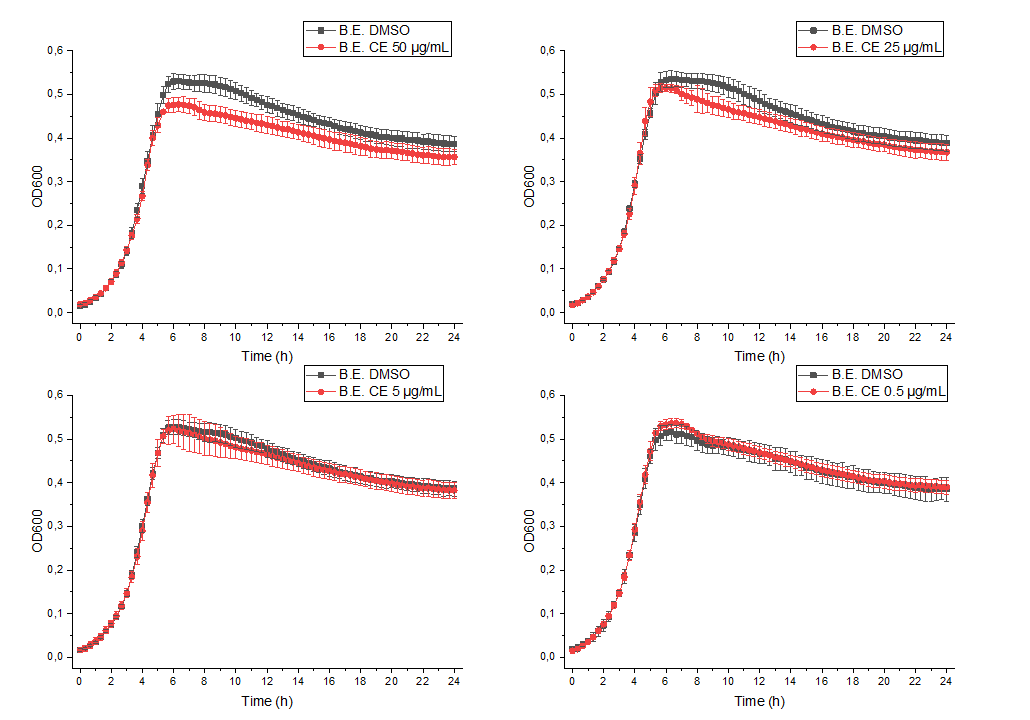


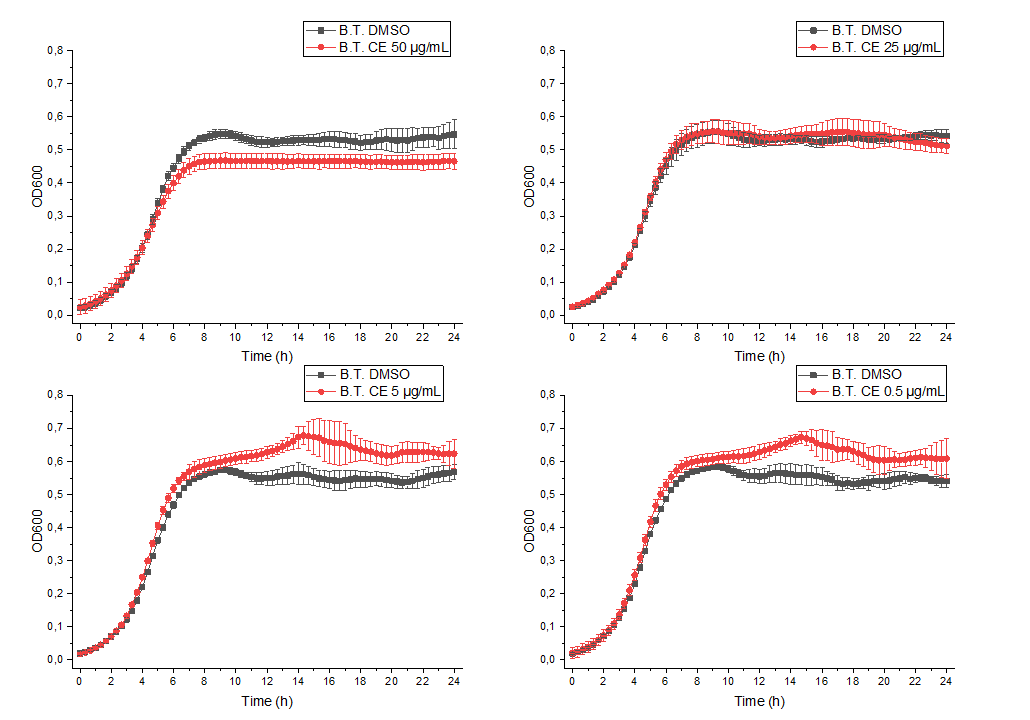


**Online Resource 3.** Continue.


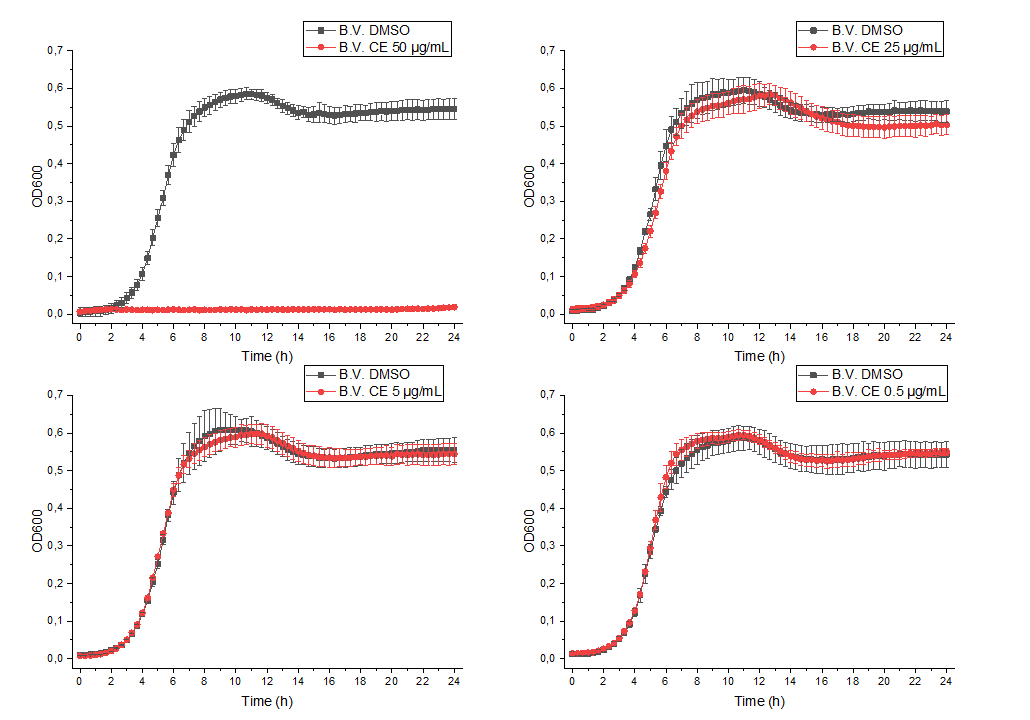


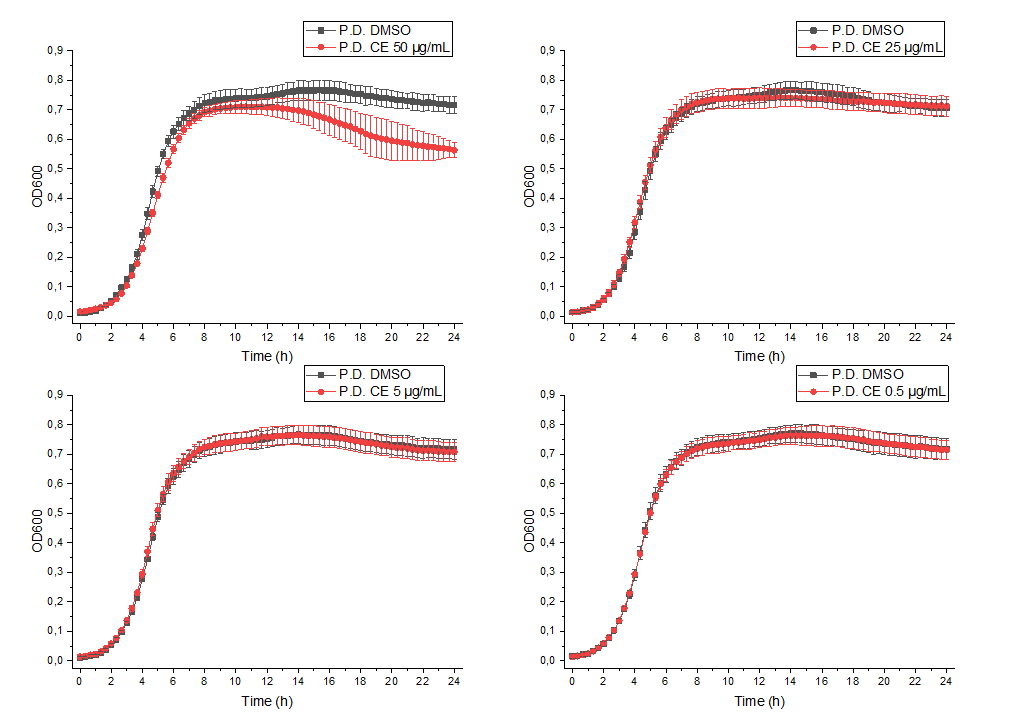


**Online Resource 3.** Continue.


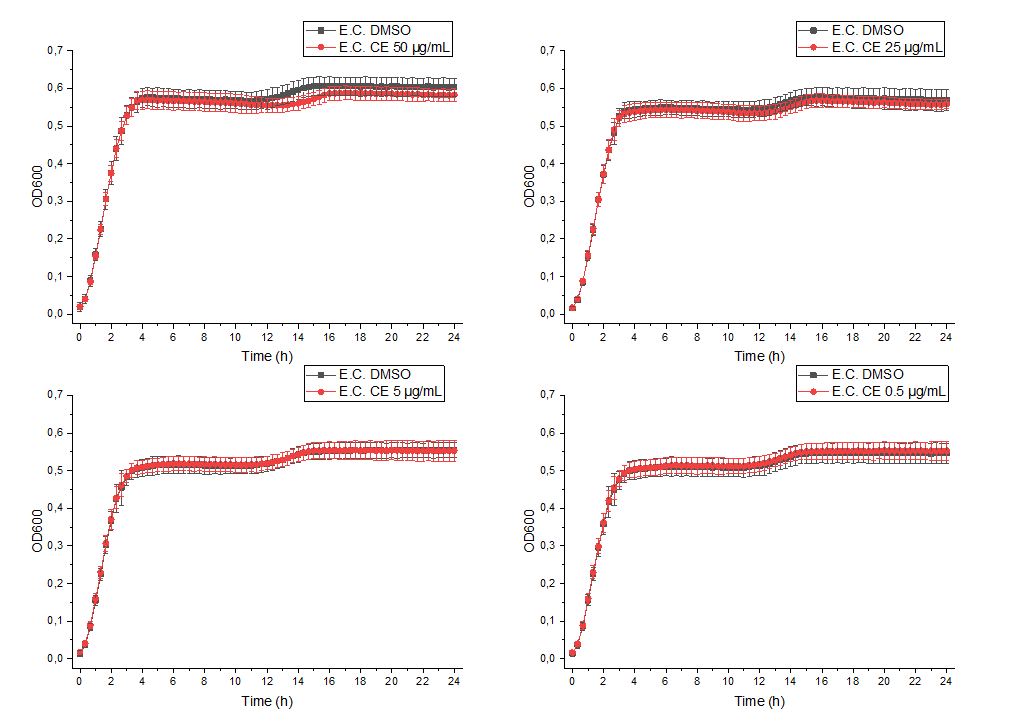


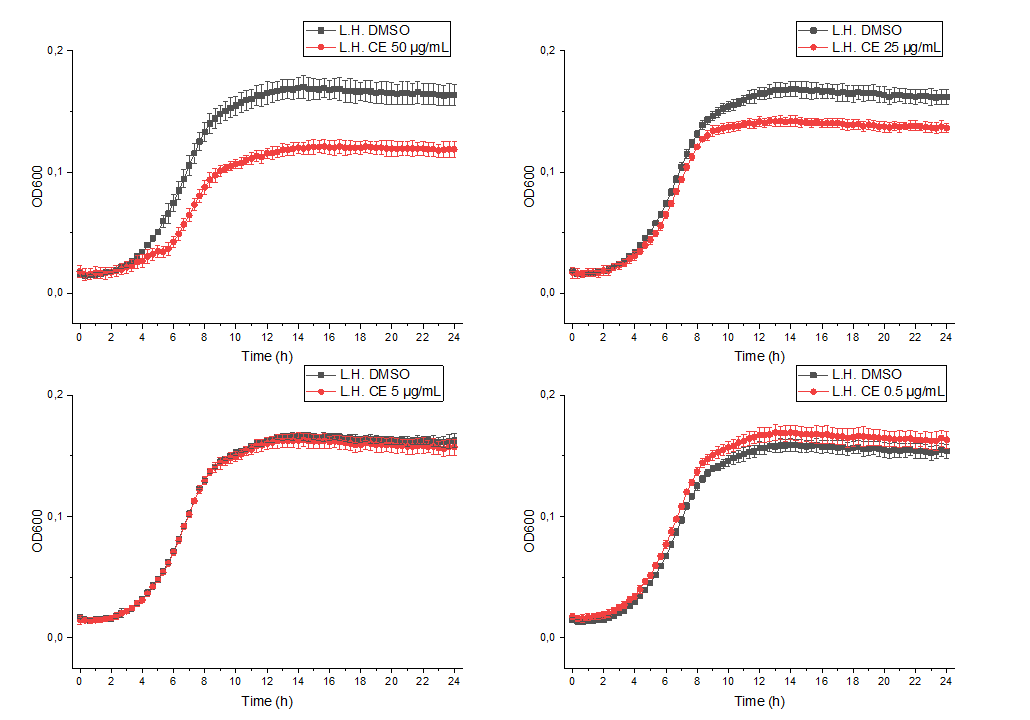


**Online Resource 3.** Continue.


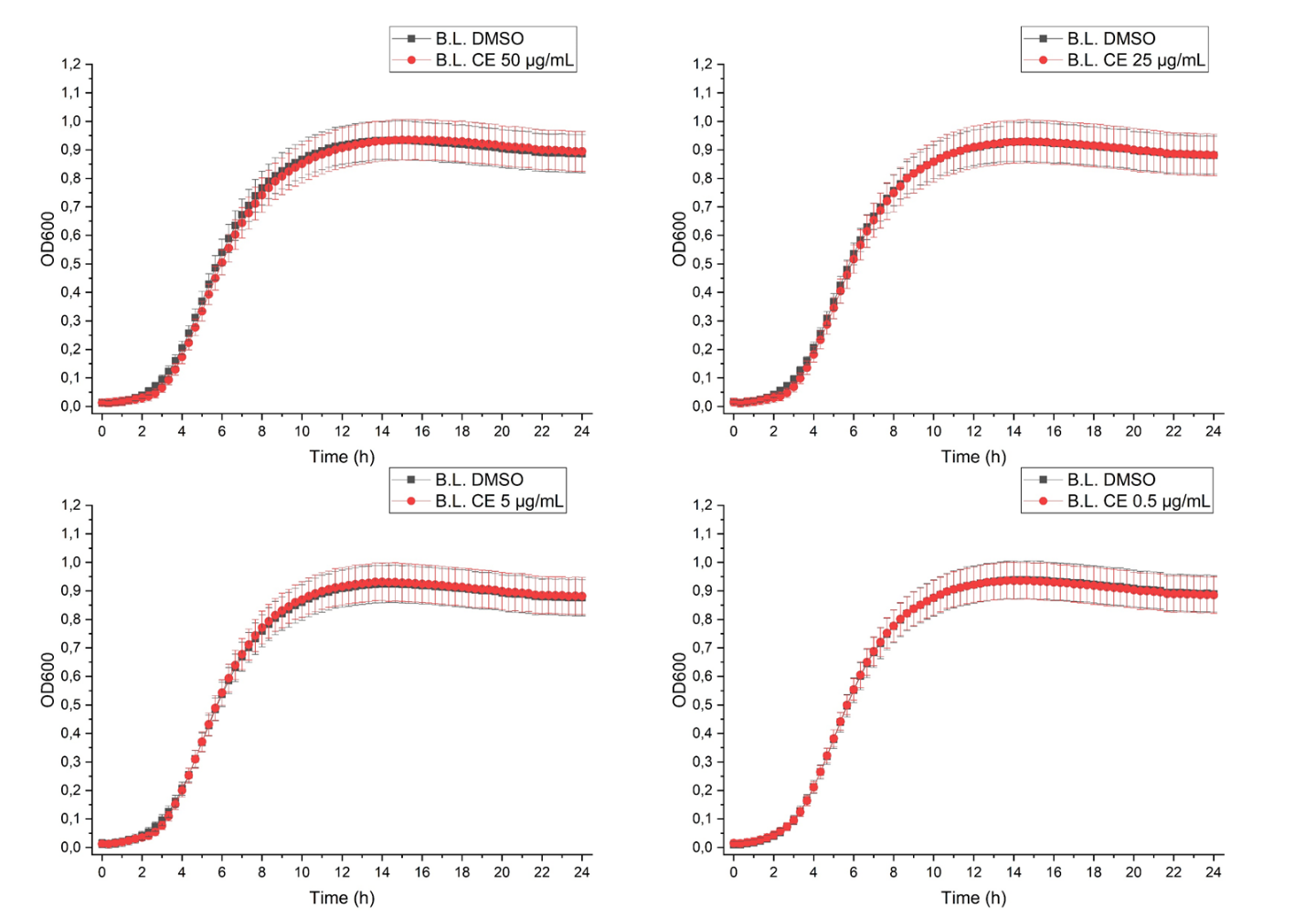


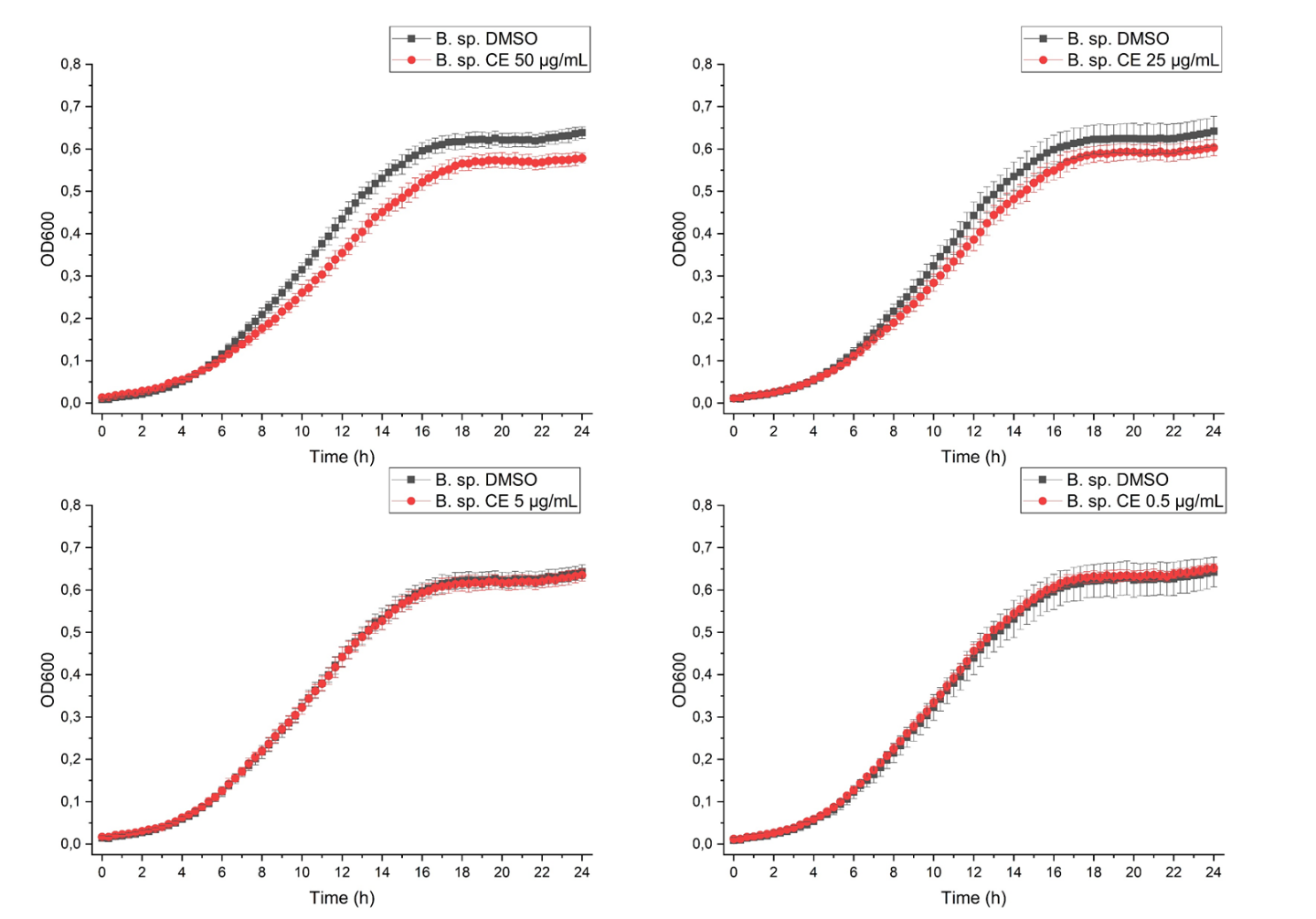


**Online Resource 3.** Continue.


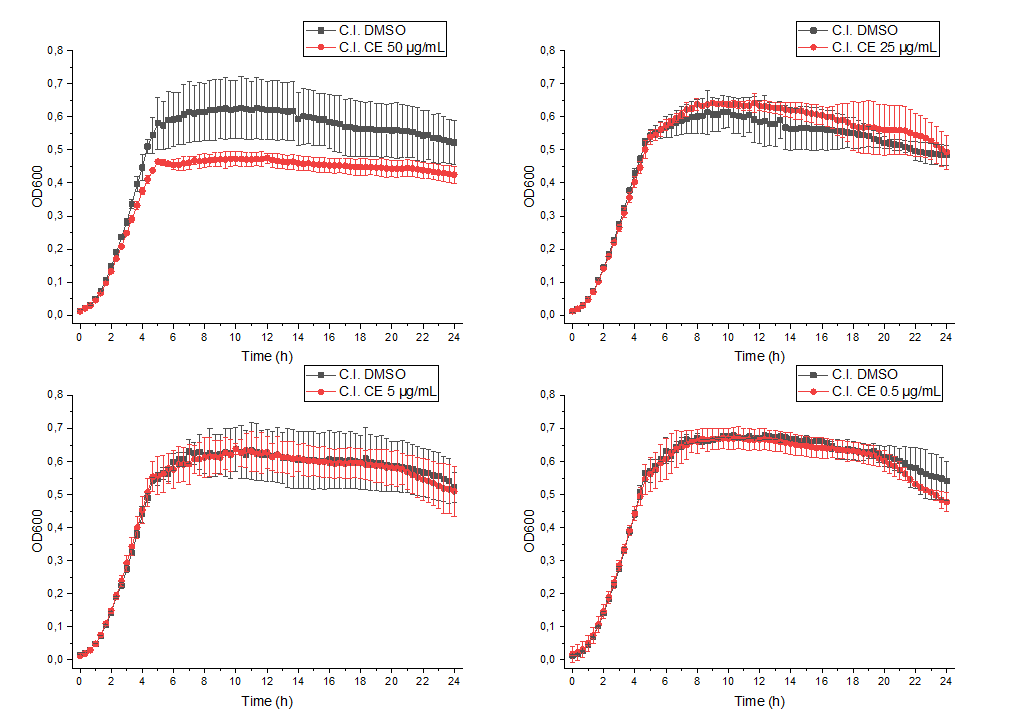


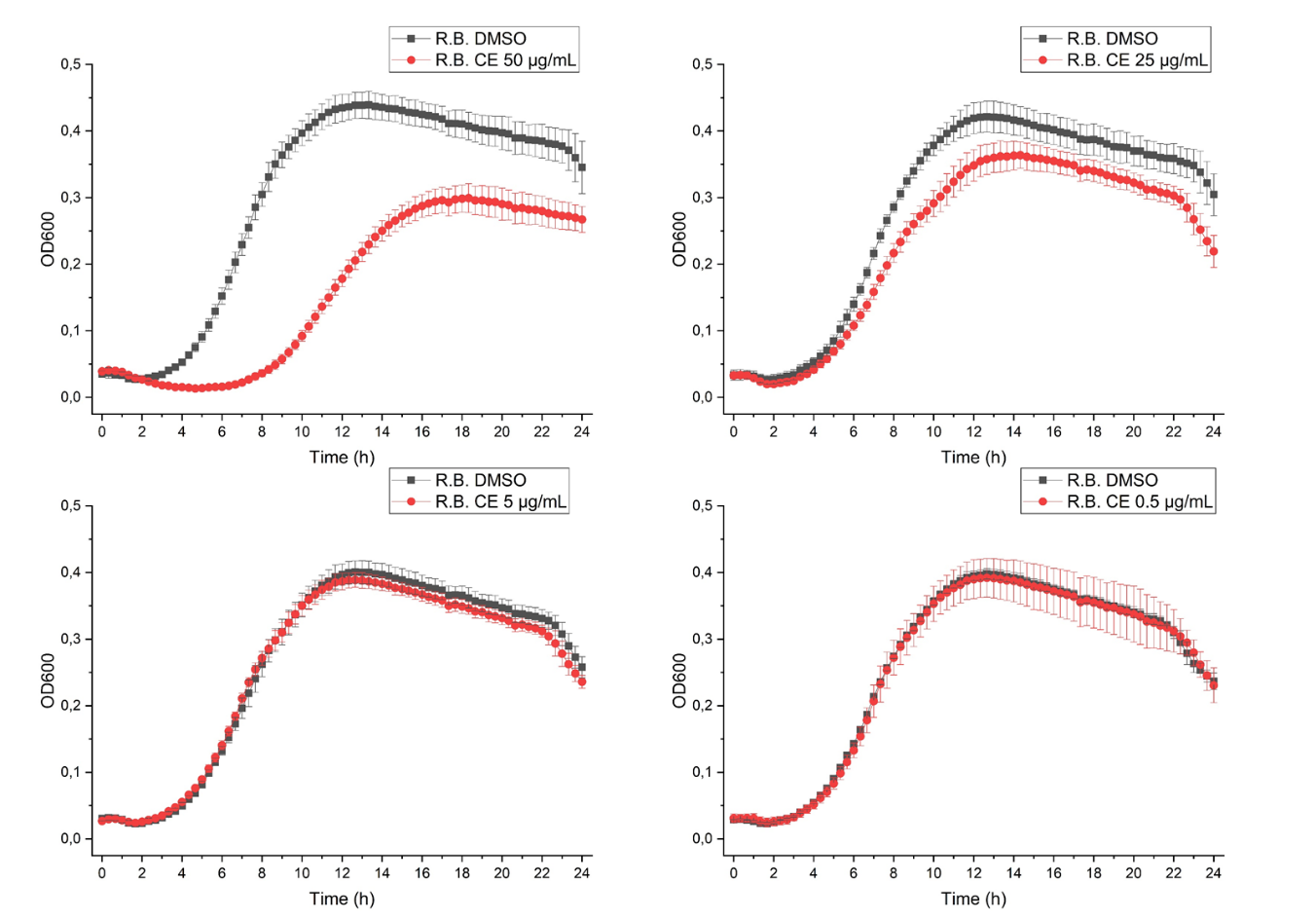


**Online Resource 3.** Continue.

# **Online Resource 4.** Doubling time of the strains exposed to the various concentrations of the *Alternaria* extract, each in comparison to the respective solvent control (0.1-0.001% DMSO).

| **Strain** |  | **Doubling time (hours; mean ± SD)** | | | | | | | | | | | | | | | | | | | | | | | | | | | | | | |
| --- | --- | --- | --- | --- | --- | --- | --- | --- | --- | --- | --- | --- | --- | --- | --- | --- | --- | --- | --- | --- | --- | --- | --- | --- | --- | --- | --- | --- | --- | --- | --- | --- |
|  |  | **Treatment with 50 µg/mL of CE** | | | | | | |  | **Treatment with 25 µg/mL of CE** | | | | | | |  | **Treatment with 5 µg/mL of CE** | | | | | | |  | **Treatment with 0.5 µg/mL of CE** | | | | | | |
|  |  | **Control** | | |  | **Treated** | | |  | **Control** | | |  | **Treated** | | |  | **Control** | | |  | **Treated** | | |  | **Control** | | |  | **Treated** | | |
| *A. finegoldii* |  | 1.03 | ± | 0.28 |  | 1.45 | ± | 0.11***** |  | 0.99 | ± | 0.05 |  | 1.24 | ± | 0.07***** |  | 1.04 | ± | 0.09 |  | 1.20 | ± | 0.05***** |  | 0.97 | ± | 0.03 |  | 1.04 | ± | 0.06 |
| *A. timonensis* |  | 2.20 | ± | 0.28 |  | 3.93 | ± | 0.73***** |  | 2.28 | ± | 0.31 |  | 2.65 | ± | 0.33 |  | 2.08 | ± | 0.12 |  | 2.10 | ± | 0.09 |  | 2.10 | ± | 0.10 |  | 2.25 | ± | 0.11 |
| *A. muciniphila* |  | 3.48 | ± | 0.44 |  | 3.87 | ± | 0.13 |  | 3.34 | ± | 0.27 |  | 3.55 | ± | 0.25 |  | 3.28 | ± | 0.26 |  | 3.61 | ± | 0.37 |  | 3.28 | ± | 0.25 |  | 3.68 | ± | 0.42 |
| *B. caccae* |  | 1.36 | ± | 0.16 |  | 1.30 | ± | 0.01 |  | 1.27 | ± | 0.11 |  | 1.28 | ± | 0.04 |  | 1.18 | ± | 0.02 |  | 1.18 | ± | 0.05 |  | 1.17 | ± | 0.09 |  | 1.18 | ± | 0.04 |
| *B. eggerthii* |  | 0.99 | ± | 0.05 |  | 1.05 | ± | 0.06 |  | 1.04 | ± | 0.03 |  | 1.03 | ± | 0.03 |  | 1.00 | ± | 0.02 |  | 1.03 | ± | 0.07 |  | 1.03 | ± | 0.04 |  | 1.03 | ± | 0.03 |
| *B. thetaiotaomicron* |  | 1.22 | ± | 0.15 |  | 1.35 | ± | 0.33 |  | 1.28 | ± | 0.02 |  | 1.27 | ± | 0.04 |  | 1.18 | ± | 0.09 |  | 1.10 | ± | 0.02 |  | 1.18 | ± | 0.08 |  | 1.13 | ± | 0.21 |
| *B. vulgatus* |  | 0.83 | ± | 0.15 |  |  | –– | ***** |  | 0.84 | ± | 0.04 |  | 1.00 | ± | 0.13 |  | 0.88 | ± | 0.03 |  | 0.82 | ± | 0.08 |  | 0.86 | ± | 0.07 |  | 0.88 | ± | 0.05 |
| *P. distasonis* |  | 0.81 | ± | 0.05 |  | 0.89 | ± | 0.03 |  | 0.83 | ± | 0.03 |  | 0.82 | ± | 0.06 |  | 0.82 | ± | 0.05 |  | 0.84 | ± | 0.06 |  | 0.86 | ± | 0.07 |  | 0.86 | ± | 0.03 |
| *E. coli* |  | 0.53 | ± | 0.05 |  | 0.53 | ± | 0.01 |  | 0.52 | ± | 0.03 |  | 0.53 | ± | 0.02 |  | 0.52 | ± | 0.03 |  | 0.54 | ± | 0.02 |  | 0.53 | ± | 0.03 |  | 0.53 | ± | 0.02 |
| *L. hominis* |  | 2.00 | ± | 0.06 |  | 2.62 | ± | 0.37***** |  | 2.07 | ± | 0.03 |  | 2.09 | ± | 0.21 |  | 1.92 | ± | 0.12 |  | 1.95 | ± | 0.10 |  | 1.96 | ± | 0.12 |  | 2.00 | ± | 0.15 |
| *B. longum* |  | 0.82 | ± | 0.15 |  | 0.78 | ± | 0.16 |  | 0.87 | ± | 0.10 |  | 0.77 | ± | 0.16 |  | 0.92 | ± | 0.12 |  | 0.84 | ± | 0.11 |  | 0.83 | ± | 0.14 |  | 0.90 | ± | 0.15 |
| *Bifidobacterium sp.* |  | 1.68 | ± | 0.03 |  | 2.13 | ± | 0,12***** |  | 1.73 | ± | 0.27 |  | 1.92 | ± | 0.02 |  | 1.72 | ± | 0.14 |  | 2.00 | ± | 0.15 |  | 1.69 | ± | 0.29 |  | 1.80 | ± | 0.10 |
| *C. innocuum* |  | 0.74 | ± | 0.04 |  | 0.80 | ± | 0.06 |  | 0.68 | ± | 0.04 |  | 0.70 | ± | 0.03 |  | 0.72 | ± | 0.05 |  | 0.67 | ± | 0.02 |  | 0.68 | ± | 0.03 |  | 0.81 | ± | 0.16 |
| *R. bicirculans* |  | 1.55 | ± | 0.06 |  | 1.61 | ± | 0.11 |  | 1.57 | ± | 0.14 |  | 1.58 | ± | 0.08 |  | 1.57 | ± | 0.05 |  | 1.62 | ± | 0.04 |  | 1.58 | ± | 0.03 |  | 1.57 | ± | 0.06 |

* Significant different compared to the respective control with DMSO. Differences were evaluated by Student’s *t*-test (p<0.05).

– No growth

# **Online Resource 5.** OD_600_ values recorded after 24 h and 48 h incubation with various concentrations of the *Alternaria* extract, each in comparison to the respective solvent control (0.1-0.001% DMSO).

| **Strain** | **Time** |  | **OD_600_ (mean ± SD)** | | | | | | | | | | | | | | | | | | | | | | | | | | | | | | |
| --- | --- | --- | --- | --- | --- | --- | --- | --- | --- | --- | --- | --- | --- | --- | --- | --- | --- | --- | --- | --- | --- | --- | --- | --- | --- | --- | --- | --- | --- | --- | --- | --- | --- |
|  |  |  | **Treatment with 50 µg/mL of CE** | | | | | | |  | **Treatment with 25 µg/mL of CE** | | | | | | |  | **Treatment with 5 µg/mL of CE** | | | | | | |  | **Treatment with 0.5 µg/mL of CE** | | | | | | |
|  |  |  | **Control** | | |  | **Treated** | | |  | **Control** | | |  | **Treated** | | |  | **Control** | | |  | **Treated** | | |  | **Control** | | |  | **Treated** | | |
| *E. coli* | 24 h |  | 0.59 | ± | 0.04 |  | 0.58 | ± | 0.03 |  | 0.59 | ± | 0.03 |  | 0.58 | ± | 0.03 |  | 0.58 | ± | 0.03 |  | 0.58 | ± | 0.04 |  | 0.58 | ± | 0.04 |  | 0.57 | ± | 0.02 |
|  | 48 h |  | 0.56 | ± | 0.04 |  | 0.56 | ± | 0.03 |  | 0.56 | ± | 0.03 |  | 0.55 | ± | 0.04 |  | 0.56 | ± | 0.02 |  | 0.56 | ± | 0.03 |  | 0.55 | ± | 0.02 |  | 0.56 | ± | 0.04 |
| *B. vulgatus* | 24 h |  | 0.59 | ± | 0.09 |  | 0.04 | ± | 0.02***** |  | 0.60 | ± | 0.07 |  | 0.52 | ± | 0.05 |  | 0.58 | ± | 0.04 |  | 0.55 | ± | 0.04 |  | 0.58 | ± | 0.06 |  | 0.63 | ± | 0.04 |
|  | 48 h |  | 0.62 | ± | 0.04 |  | 0.02 | ± | 0.01***** |  | 0.63 | ± | 0.08 |  | 0.56 | ± | 0.04 |  | 0.60 | ± | 0.03 |  | 0.58 | ± | 0.06 |  | 0.61 | ± | 0.03 |  | 0.60 | ± | 0.06 |
| *B. thetaiotaomicron* | 24 h |  | 0.64 | ± | 0.04 |  | 0.63 | ± | 0.03 |  | 0.64 | ± | 0.03 |  | 0.63 | ± | 0.04 |  | 0.62 | ± | 0.04 |  | 0.63 | ± | 0.03 |  | 0.62 | ± | 0.03 |  | 0.63 | ± | 0.03 |
|  | 48 h |  | 0.56 | ± | 0.03 |  | 0.56 | ± | 0.03 |  | 0.56 | ± | 0.04 |  | 0.56 | ± | 0.03 |  | 0.56 | ± | 0.04 |  | 0.56 | ± | 0.04 |  | 0.56 | ± | 0.03 |  | 0.55 | ± | 0.03 |
| *B. caccae* | 24 h |  | 0.66 | ± | 0.05 |  | 0.64 | ± | 0.04 |  | 0.65 | ± | 0.02 |  | 0.64 | ± | 0.03 |  | 0.65 | ± | 0.03 |  | 0.66 | ± | 0.04 |  | 0.67 | ± | 0.03 |  | 0.65 | ± | 0.04 |
|  | 48 h |  | 0.66 | ± | 0.04 |  | 0.61 | ± | 0.04 |  | 0.67 | ± | 0.06 |  | 0.61 | ± | 0.03 |  | 0.63 | ± | 0.02 |  | 0.65 | ± | 0.03 |  | 0.72 | ± | 0.03 |  | 0.66 | ± | 0.03 |

* Significant different compared to control with DMSO. Differences were evaluated by Student’s *t*-test (p<0.05).

# **Online Resource 6.** Total recovery of *Alternaria* mycotoxins after 24 h incubation of strains with 25 µg/mL of CE

| Strain | AOH | | |  | AME | | |  | ALT | | |  | TeA | | |  | TEN | | |  | ATX-I | | |  | ALP | | |  | ALS | | |  | AST | | |  |
| --- | --- | --- | --- | --- | --- | --- | --- | --- | --- | --- | --- | --- | --- | --- | --- | --- | --- | --- | --- | --- | --- | --- | --- | --- | --- | --- | --- | --- | --- | --- | --- | --- | --- | --- | --- | --- |
| *A. finegoldii* | 52.0* | ± | 5.0 |  | 69.3* | ± | 2.1 |  | 120 | ± | 18 |  | 103 | ± | 5 |  | 83.0 | ± | 44.6 |  | 106* | ± | 35 |  | 34.8* | ± | 8.8 |  | 102* | ± | 15 |  | 30.6* | ± | 2.5 |  |
| *A. timonensis* | 69.3* | ± | 19.2 |  | 69.6* | ± | 10.1 |  | 127 | ± | 8 |  | 103 | ± | 6 |  | 114 | ± | 37 |  | 100 | ± | 28 |  | 64.5* | ± | 26.7 |  | 87.5 | ± | 8.8 |  | 57.6* | ± | 14.6 |  |
| *A. muciniphila* | 53.7* | ± | 10.8 |  | 92.9 | ± | 13.3 |  | 106 | ± | 16 |  | 86.6 | ± | 5.6 |  | 58.1* | ± | 12.1 |  | 100 | ± | 3 |  | 56.4* | ± | 1.6 |  | 90.8 | ± | 4.2 |  | 60.0* | ± | 8.5 |  |
| *B. caccae* | 61.6* | ± | 15.3 |  | 75.0* | ± | 19.0 |  | 133 | ± | 25 |  | 109 | ± | 11 |  | 86.8 | ± | 10.9 |  | 138 | ± | 43 |  | 24.7* | ± | 7.6 |  | 83.5 | ± | 10.8 |  | 70.5* | ± | 17.2 |  |
| *B. eggerthii* | 118 | ± | 11 |  | 72.4* | ± | 10.0 |  | 63.1 | ± | 33.9 |  | 100 | ± | 12 |  | 122 | ± | 16 |  | 142* | ± | 2 |  | 57.9* | ± | 6.7 |  | 93.1 | ± | 13.3 |  | 64.3* | ± | 6.3 |  |
| *B. thetaiotaomicron* | 42.3* | ± | 2.5 |  | 77.3* | ± | 18.0 |  | 91.6 | ± | 25.1 |  | 106 | ± | 7 |  | 102 | ± | 24 |  | 133 | ± | 49 |  | 20.3* | ± | 4.6 |  | 92.4 | ± | 12.7 |  | 61.9* | ± | 15.0 |  |
| *B. vulgatus* | 58.1* | ± | 11.5 |  | 91.4* | ± | 2.3 |  | 100 | ± | 38 |  | 92.5 | ± | 8.5 |  | 85.5 | ± | 37.5 |  | 89.8 | ± | 1.7 |  | 24.4* | ± | 5.3 |  | 98.7 | ± | 13.0 |  | 74.5* | ± | 11.7 |  |
| *P. distasonis* | 80.2* | ± | 3.4 |  | 73.2* | ± | 4.7 |  | 84.4 | ± | 37.8 |  | 96.9 | ± | 6.4 |  | 116 | ± | 12 |  | 183 | ± | 4 |  | 26.3* | ± | 2.5 |  | 86.6 | ± | 4.9 |  | 50.7* | ± | 16.7 |  |
| *E. coli* | 33.5* | ± | 4.4 |  | 55.4* | ± | 12.0 |  | 119 | ± | 12 |  | 103 | ± | 11 |  | 93.0 | ± | 24.5 |  | 93.7 | ± | 10.4 |  | 31.0* | ± | 1.7 |  | 93.5 | ± | 10.2 |  | 48.7* | ± | 6.9 |  |
| *L. hominis* | 60.7* | ± | 20.3 |  | 87.2 | ± | 29.5 |  | 77.2 | ± | 8.9 |  | 125 | ± | 13 |  | 69.5 | ± | 19.4 |  | 93.8 | ± | 33.5 |  | 101 | ± | 36 |  | 56.1* | ± | 8.1 |  | 68.3* | ± | 4.6 |  |
| *B. longum* | 46.1* | ± | 6.0 |  | 102 | ± | 28 |  | 65.0 | ± | 20.9 |  | 118 | ± | 8 |  | 80.5 | ± | 7.6 |  | 80.8* | ± | 2.5 |  | 75.0* | ± | 1.6 |  | 49.9* | ± | 4.6 |  | 73.2* | ± | 10.2 |  |
| *Bifidobacterium sp.* | 102 | ± | 16 |  | 75.3* | ± | 8.6 |  | 53.8* | ± | 7.0 |  | 119 | ± | 3 |  | 62.0* | ± | 4.8 |  | 136* | ± | 6 |  | 117* | ± | 2 |  | 51.4* | ± | 8.9 |  | 56.8* | ± | 4.8 |  |
| *C. innocuum* | 50.1* | ± | 4.2 |  | 84.5* | ± | 10.3 |  | 120 | ± | 3 |  | 96.3 | ± | 6.4 |  | 79.7 | ± | 9.8 |  | 119 | ± | 31 |  | 19.0* | ± | 5.0 |  | 93.6 | ± | 15.7 |  | 87.7 | ± | 20.0 |  |
| *R. bicirculans* | 80.8 | ± | 17.4 |  | 90.4 | ± | 14.1 |  | 112 | ± | 12 |  | 89.4 | ± | 8.7 |  | 79.0 | ± | 8.4 |  | 131 | ± | 43 |  | 14.0* | ± | 3.3 |  | 86.4 | ± | 24.8 |  | 91.9 | ± | 33.4 |  |

* Significant different compared to control with DMSO. Differences were evaluated by Student’s *t*-test (p<0.05).

.
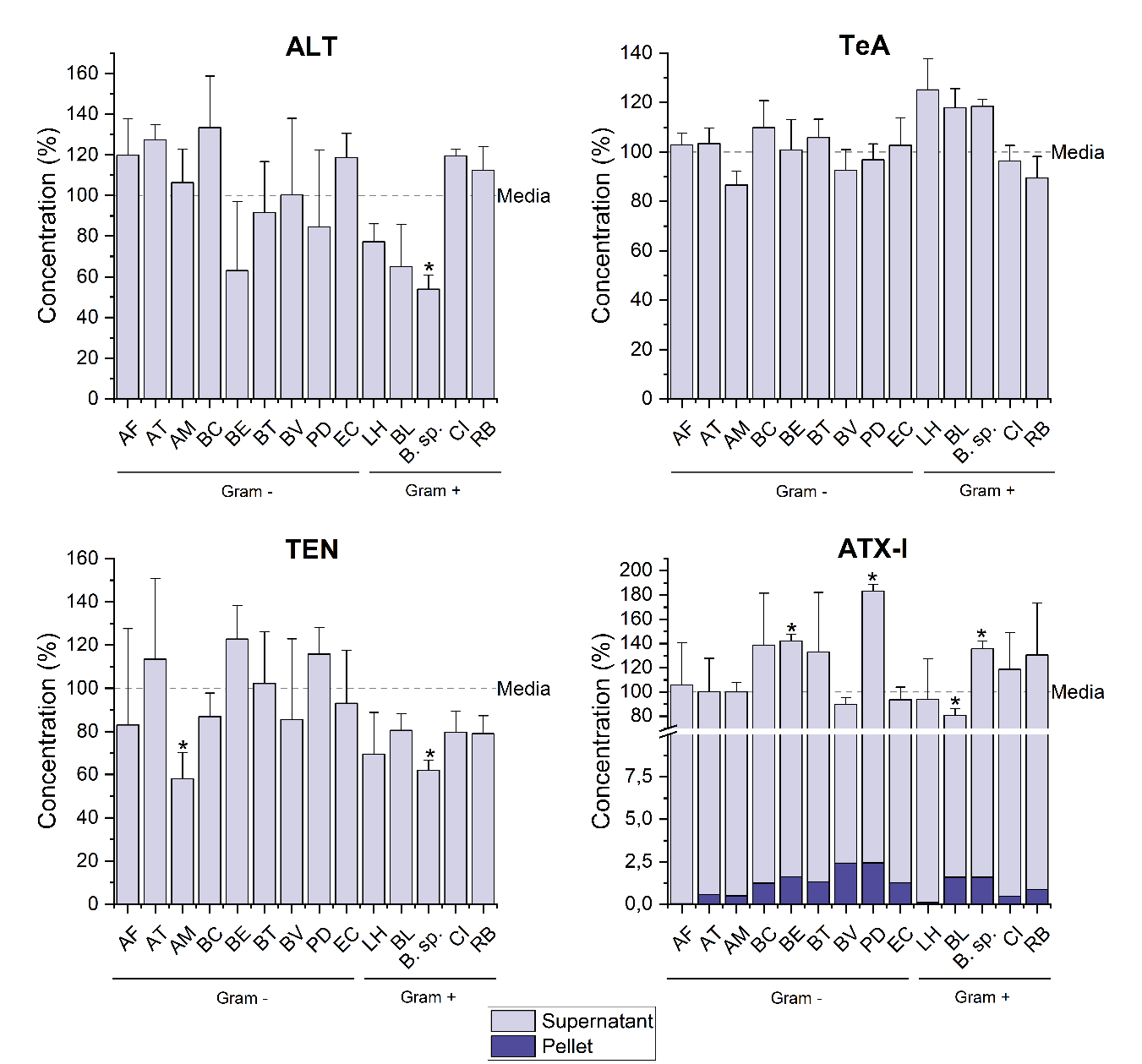


# **Online Resource 7.** Bar charts showing the amount of the least affected mycotoxins recovered in pellets and supernatants of the tested strains after 24 h incubation with 25 µg/mL of CE. Data are reported as means + SD. Differences between the total mycotoxin recovery in samples and media controls (media + CE) were evaluated by Student’s *t*-test (* = p<0.05).

AF: *A. finegoldii*; AT: *A. timonensis*; AM: *A. muciniphila*; BC: *B. caccae*; BE: *B. eggerthii*; BT: *B. thetaiotaomicron*; BV: *B. vulgatus*; PD: *P. distasonis*; EC: *E. coli*; LH: *L. hominis*; BL: *B. longum*; B. sp.: *Bifidobacterium sp.*; CI: *C. innocuum*; RB: *R. bicirculans*.


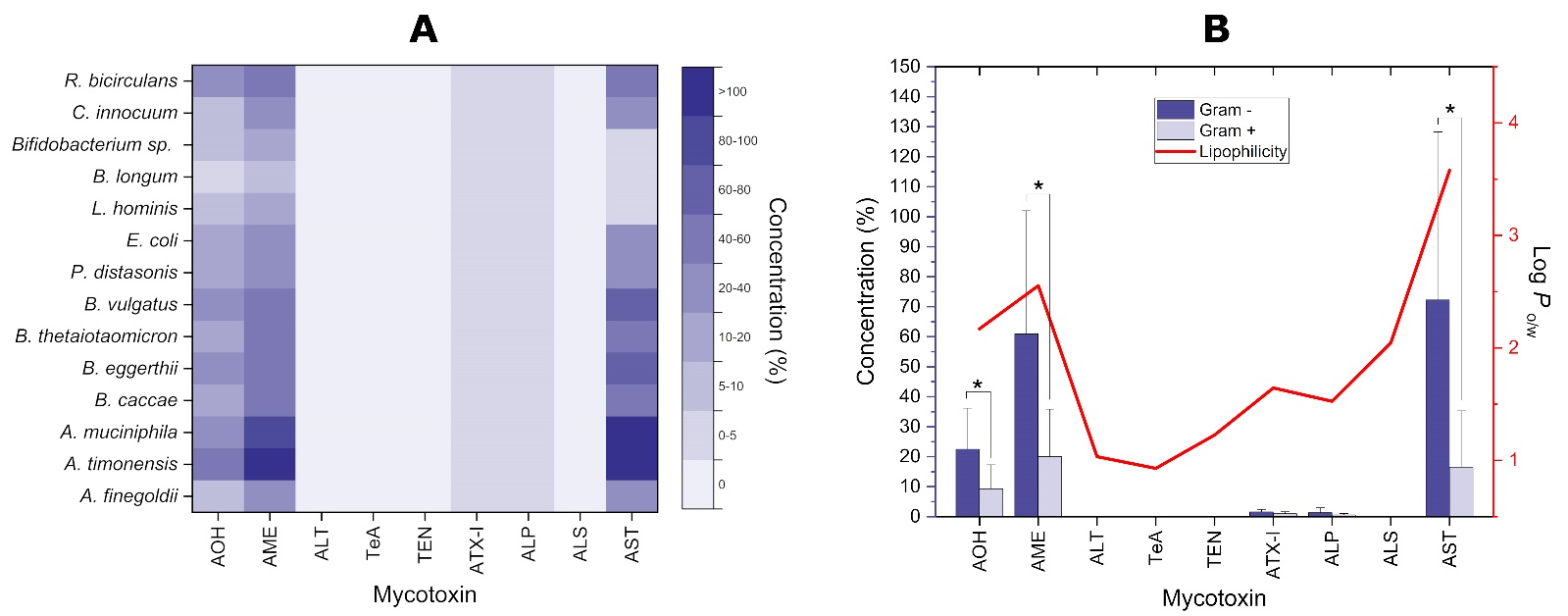


# **Online Resource 8** Theoretical recoveries of mycotoxins from bacterial pellets. Theoretical recoveries were calculated by normalizing the experimentally determined mycotoxin concentrations in bacterial pellets to a theoretical OD600 value of 0.5, corresponding to the median OD600 value recorded in the suspensions containing the various strains tested. (a) Heatmap showing the theoretical average amount (in % compared to the total mycotoxin recovery experimentally determined) of mycotoxins in bacterial pellets after 24 h incubation with 25 µg/mL of CE. (b) Double-axis plot showing the theoretical mean ± SD (in % compared to the total mycotoxin recovery experimentally determined) of mycotoxin concentrations in pellets of Gram negative and positive strains (blue and light blue columns, respectively; left axis), and the mean value of theoretical lipophilicity (solid line; right axis) of mycotoxins. Significant differences between Gram negative and positive strains were evaluated by Student’s *t*-test (*p < 0.05).

# **Online Resource 9.** Concentrations of mycotoxins the bacterial strains were exposed to during treatments with various concentrations of the *Alternaria* extract.

| Mycotoxins |  | Mycotoxin concentrations (nM) per treatment^a^ | | | | | | |
| --- | --- | --- | --- | --- | --- | --- | --- | --- |
|  |  | 50 µg/mL |  | 25 µg/mL |  | 5 µg/mL |  | 0.5 µg/mL |
| AOH |  | 153 |  | 76.5 |  | 15.3 |  | 1.53 |
| AME |  | 119 |  | 59.7 |  | 11.9 |  | 1.19 |
| ALT |  | 133 |  | 66.7 |  | 13.3 |  | 1.33 |
| TeA |  | 151599 |  | 75799 |  | 15160 |  | 1516 |
| TEN |  | 2.41 |  | 1.21 |  | 0.24 |  | 0.02 |
| ATX-I |  | 1407 |  | 704 |  | 141 |  | 14.1 |
| ATX-II |  | 2012 |  | 1006 |  | 201 |  | 20.1 |
| ALP |  | 1798 |  | 899 |  | 180 |  | 18.0 |
| STTX-III |  | 3014 |  | 1507 |  | 301 |  | 30.1 |
| ALS |  | 48.2 |  | 24.1 |  | 4.82 |  | 0.48 |
| AST |  | 2300 |  | 1150 |  | 230 |  | 23.0 |

^a^ Mycotoxin concentrations the bacterial strains were exposed to were calculated based on the concentrations experimentally determined by Puntscher *et al.* (2019b)
